# Follow-up of the GHSG HD16 trial of PET-guided treatment in early-stage favorable Hodgkin lymphoma

**DOI:** 10.1038/s41375-023-02064-y

**Published:** 2023-10-16

**Authors:** Michael Fuchs, Anne Sophie Jacob, Helen Kaul, Carsten Kobe, Georg Kuhnert, Thomas Pabst, Richard Greil, Paul J. Bröckelmann, Max S. Topp, Marianne Just, Bernd Hertenstein, Martin Soekler, Martin Vogelhuber, Josée M. Zijlstra, Ulrich Bernd Keller, Stefan W. Krause, Ulrich Dührsen, Julia Meissner, Andreas Viardot, Hans-Theodor Eich, Christian Baues, Volker Diehl, Andreas Rosenwald, Ina Buehnen, Bastian von Tresckow, Markus Dietlein, Peter Borchmann, Andreas Engert, Dennis A. Eichenauer

**Affiliations:** 1 German Hodgkin Study Group (GHSG), Department I of Internal Medicine, Center for Integrated Oncology Aachen Bonn Cologne Duesseldorf, University of Cologne, Cologne, Germany; 2 Department of Nuclear Medicine, University of Cologne, Cologne, Germany; 3 Nuclear Medicine Siegen I Kreuztal, Siegen, Germany; 4 Department of Medical Oncology, Inselspital, Bern University Hospital, University of Bern, Bern, Switzerland; 5 Swiss Group for Clinical Cancer Research (SAKK), Bern, Switzerland; 6 IIIrd Medical Department, Paracelcus Medical University and Salzburg Cancer Research Institute, Salzburg, Austria; 7 Salzburg Cancer Research Institute and AGMT (Arbeitsgemeinschaft Medikamentöse Tumortherapie), Salzburg, Austria; 8 Medizinische Klinik und Poliklinik II, Universitätsklinikum Würzburg, Würzburg, Germany; 9 Dres. med. Just/Düwel/Riesenberg/Steinke/Schäfer, Studiengesellschaft, Bielefeld, Germany; 10 Department of Internal Medicine I, Klinikum Bremen Mitte, Bremen, Germany; 11 Onkology, Spital Thun, Switzerland, formerly University of Tübingen, Tübingen, Germany; 12 Medizinische Klinik III, Universitätsklinik Regensburg, Regensburg, Germany; 13 Department of Hematology, Amsterdam UMC, Vrije Universiteit, Amsterdam, Netherlands; 14 Department of Internal Medicine III, Klinikum “Rechts der Isar”, Munich, Germany; 15 Department of Internal Medicine 5, Haematology/Oncology, Uniklinikum Erlangen, Erlangen, Germany; 16 Department of Haematology, University Hospital Essen, University of Duisburg-Essen, Essen, Germany; 17 University of Heidelberg, Heidelberg, Germany; 18 Department of Internal Medicine III, University Hospital Ulm, Ulm, Germany; 19 Department of Radiotherapy, University Hospital of Muenster, Muenster, Germany; 20 Department of Radiotherapy, University of Cologne, Cologne, Germany; 21 Institute of Pathology, Julius Maximilian University of Würzburg and Comprehensive Cancer Center Mainfranken, Würzburg, Germany; 22 Department of Haematology and Stem Cell Transplantation, West German Cancer Center and German Cancer Consortium (DKTK partner site Essen), University Hospital Essen, University of Duisburg-Essen, Essen, Germany

**Keywords:** Chemotherapy, Radiotherapy

## Abstract

The primary analysis of the GHSG HD16 trial indicated a significant loss of tumor control with PET-guided omission of radiotherapy (RT) in patients with early-stage favorable Hodgkin lymphoma (HL). This analysis reports long-term outcomes. Overall, 1150 patients aged 18–75 years with newly diagnosed early-stage favorable HL were randomized between standard combined-modality treatment (CMT) (2x ABVD followed by PET/CT [PET-2] and 20 Gy involved-field RT) and PET-2-guided treatment omitting RT in case of PET-2 negativity (Deauville score [DS] < 3). The study aimed at excluding inferiority of PET-2-guided treatment and assessing the prognostic impact of PET-2 in patients receiving CMT. At a median follow-up of 64 months, PET-2-negative patients had a 5-year progression-free survival (PFS) of 94.2% after CMT (*n* = 328) and 86.7% after ABVD alone (*n* = 300; HR = 2.05 [1.20–3.51]; *p* = 0.0072). 5-year OS was 98.3% and 98.8%, respectively (*p* = 0.14); 4/12 documented deaths were caused by second primary malignancies and only one by HL. Among patients assigned to CMT, 5-year PFS was better in PET-2-negative (*n* = 353; 94.0%) than in PET-2-positive patients (*n* = 340; 90.3%; *p* = 0.012). The difference was more pronounced when using DS4 as cut-off (DS 1-3: *n* = 571; 94.0% vs. DS ≥ 4: *n* = 122; 83.6%; *p* < 0.0001). Taken together, CMT should be considered standard treatment for early-stage favorable HL irrespective of the PET-2-result.

## Introduction

Since long-term remission rates for Hodgkin lymphoma (HL) patients with early-stage favorable disease are already above 90% after combined modality first-line therapy (CMT) [1–5], research is putting more emphasis on improving the balance between long-term side effects on the one hand and tumor control on the other [6].

A widely used standard of care for early-stage favorable HL is treatment with two cycles of doxorubicin, bleomycin, vinblastine, and dacarbazine (ABVD) followed by 20 Gy involved-site radiotherapy (RT). However, the RT consolidation within the CMT concept is assumed to be more harmful than the chemotherapy [7–10]. Therefore, several trials have assessed whether the use of positron emission tomography (PET) may allow to omit RT from the standard regimen, including the German Hodgkin Study Group (GHSG) HD16 trial [10–13]. In HD16, we aimed at demonstrating non-inferiority when omitting consolidation RT in patients with a negative PET after two cycles of ABVD (PET-2) in terms of progression-free survival (PFS) as compared to CMT. Secondly, we analyzed whether a positive PET-2 was a risk factor for PFS among patients who were treated with CMT. Here, we present the follow-up analysis on the final data status reporting the long-term results of the international randomized GHSG phase III HD16 trial.

## Methods

### Study design and patients

The randomized phase 3 trial HD16 was conducted at 250 sites in Germany, Switzerland, Austria, and the Netherlands. It was approved by the responsible ethics committees, conducted according to the Declaration of Helsinki and the ICH-GCP guidelines, registered at ClinicalTrials.gov (NCT00736320), and completed enrollment in December 2015 [13]. All patients provided written informed consent before enrollment.

We recruited patients aged 18–75 years with newly diagnosed, histology-proven classical HL in clinical stages I or II, or nodular lymphocyte-predominant HL in Ann Arbor stages IB, IIA, or IIB, without any of the GHSG risk factors large mediastinal mass (≥a third of the maximal thoracic diameter), extranodal lesions, elevated erythrocyte sedimentation rate (≥50 mm/h without B symptoms, ≥30 mm/h with B symptoms), or ≥3 involved nodal areas. Further details of study design and inclusion/exclusion criteria have been previously published [13].

### Randomization

Patients were centrally randomized 1:1 between two parallel treatment groups before starting treatment: CMT consisting of two cycles of ABVD followed by a centrally reviewed PET/CT-based staging (PET-2) and 20 Gy IF-RT or PET-2-guided treatment, with two cycles of ABVD for all patients and 20 Gy IF-RT only for those with a positive PET-2 as defined by a Deauville score (DS) ≥ 3. Randomization was stratified according to recruiting site, age (<45 vs. ≥45 years), sex, B symptoms, disease localization (supradiaphragmatic vs infradiaphragmatic), albumin level (<40 g/L vs ≥40 g/L), and presence vs. absence of initial bulk ≥5 cm in largest diameter. Patients, investigators, and central review panel were masked to treatment allocation until a central review of PET-2 was completed.

### Procedures

Procedures have been published in detail [13] and are summarized in the supplement. ABVD was given as previously described [10]. PET-2 was performed between day 22 and 35 of the second ABVD cycle, centrally reviewed by a multidisciplinary panel of experts masked to treatment group allocation and rated according to the DS using the mediastinal blood pool as cut-off for positivity (DS ≥ 3) [14]. Patients with progressive disease were to be taken off study treatment. IF-RT was centrally planned for all patients based on initial staging imaging [15].

Patients were followed for at least 5 years within the study. If separate written informed consent was given, individual follow-up was extended until the end of the study, scheduled 5 years after enrollment of the last patient.

### Outcomes

The primary endpoint was PFS, defined as time from completion of staging until progression, relapse, or death from any cause. If none of these events had occurred, PFS was censored at the date of last information on the disease status. Predefined secondary endpoints reported herein were overall survival (OS; time from completion of staging until death from any cause or censored at the date of last information on the patient being alive), time to second primary malignancy (SPM; calculated from completion of staging until first SPM diagnosis or censored at the date of last information on disease status, accounting for death as a competing risk), time to in-field and out-field recurrence, with in-field recurrence defined as progression or relapse with at least one localization within the (potential) radiation field, and out-field recurrences as those with at least one localization outside of the (potential) radiation field (calculated from completion of staging until first respective event or censored at the date of last information on disease status, accounting for death or other HL recurrence as competing risks), cardiac function in terms of mean left ventricular ejection fraction (LVEF) after 5 years of follow-up, time to first childbirth (calculated in female patients from last day of study therapy until the day of birth of the first child born after study therapy, or censored at the date of last information on disease status, accounting for death as a competing risk), as well as frequency of children born after therapy and use of cryopreservation.

### Statistical analysis

The HD16 trial had two independent co-primary objectives. The first question addressed whether RT could be omitted from standard CMT after a negative PET-2 without a clinically relevant loss of tumor control. Non-inferiority of ABVD alone over standard CMT would be established if the upper limit of the 2-sided 95% CI for the hazard ratio (HR) for PFS was below the predefined non-inferiority margin of 3.01 in a per-protocol analysis of the PET-2-negative patient population. Second, the HD16 trial aimed to assess whether a positive PET-2 represented a risk factor for PFS among patients assigned to receive CMT by demonstrating superiority of patients with a negative PET-2 result from the standard CMT group over those with a positive PET-2 from both treatment groups. Details on statistical methods and sample size calculation have been published previously [13].

OS as well as in-field and out-field recurrences, were analyzed as secondary endpoints for the before mentioned RT and PET objectives. SPM were analyzed in the PET-2-negative population to complement the primary non-inferiority objective. Cardiac function was analyzed in the entire study population by assigned treatment group separately for male and female patients in a complete-case analysis. Fertility outcomes were analyzed by assigned treatment groups in pre-defined subgroups of male patients aged 18–60 years at enrollment and female patients aged 18–40 years at enrollment.

We analyzed PFS and OS using the Kaplan-Meier method, including HRs and 95% CIs obtained from Cox regression models and log-rank tests where applicable. To assess the prognostic impact of PET-2 independently from baseline factors, this analysis was performed by multivariate Cox regression including all stratification factors (except for recruiting site). SPM, in-field and out-field recurrences and childbirth after therapy were analyzed using cumulative incidence functions, with sub-distribution HRs and p values obtained from univariate Fine-Gray models. Other secondary endpoints were analyzed by means of descriptive statistics. Analysis sets remained unchanged from the primary analysis [13]. The non-inferiority test was primarily performed in the per-protocol population, excluding all patients with severe protocol deviations. Sensitivity analyses and all other analyses were done by intention-to-treat. However, all patients dropping out before the central review of PET-2 were excluded from analyses regarding the main objectives of the trial (ITT_PET_ population). We did post-hoc subgroup analyses of female patients, male patients, and patients below the age of 50 years at enrollment for the non-inferiority objective; results are reported in Supplementary Fig. 2. All authors had access to primary clinical trial data. Data were analyzed in the GHSG Trial Coordination Center. We used SAS version 9.4 for all analyses. All analyses are based on the final data status of July 2021.

### Data sharing

The datasets generated and analyzed during the study and single patient data can be made available upon reasonable request. Decisions regarding data sharing will be made on a case-by-case basis by the corresponding author considering data protection and other applicable regulations.

## Results

**Fig. 1:**
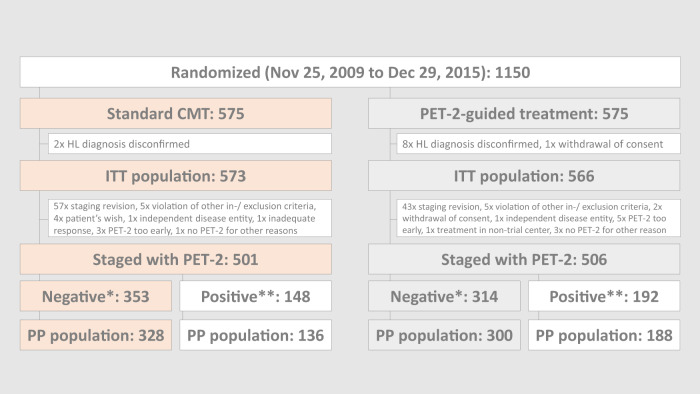
Trial profile. Abbreviations: CMT combined-modality treatment, PET-2 positron emission tomography after two cycles of chemotherapy, HL Hodgkin lymphoma, ITT intention-to-treat, PP per-protocol. *Deauville score 1–2. **Deauville score ≥3.

A total of 1150 patients were enrolled and randomized between November 25, 2009, and December 29, 2015 (Fig. 1). Among 1007 patients with a centrally reviewed PET-2, 340 were rated positive, with DS 3 in 218, DS 4 in 122, and DS 5 in 0 cases. Among the 667 patients with a negative PET-2, the per-protocol set comprises 628 patients, 328 of which were treated with standard CMT and 300 with ABVD alone. Patient characteristics have been published [13] and were similar between randomized treatment groups in the ITT_PET_ as well as the PET-2-negative per-protocol population (Supplementary Tables 1–2).

**Fig. 2:**
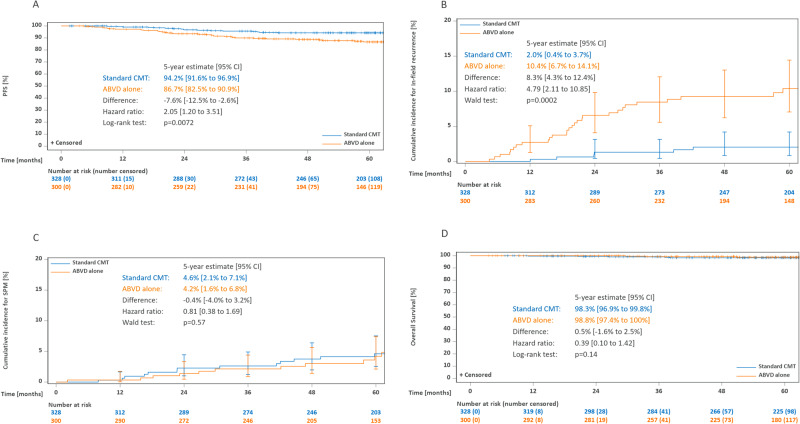
Kaplan-Meier estimates for the PET-2-negative per-protocol population. **A** Progression-free survival. **B** Cumulative incidence of in-field recurrences. **C** Cumulative incidence of second primary malignancies. **D** Overall survival. Abbreviations: PFS progression-free survival, CMT combined-modality treatment, ABVD doxorubicin, bleomycin, vinblastine, and dacarbazine, SPM second primary malignancy.

With a median follow-up of 64 months, estimated 5-year PFS in the PET-2-negative per-protocol population was 94.2% (91.6–96.9) with CMT and 86.7% (82.5–90.9) with ABVD alone (Fig. 2A). With a HR of 2.05, the respective 95% CI ranged from 1.20 to 3.51 and thus included the pre-defined margin for non-inferiority of 3.01. The ITT analysis led to similar results (5-year PFS 94.0% [91.4–96.6] with CMT and 86.6% [82.4–90.7] with ABVD alone, HR 1.99 [1.19–3.34], Supplementary Fig. 1A). The PFS difference primarily resulted from an incidence of in-field recurrences in the ABVD only group, with 5-year cumulative incidences of 2.0% (0.4–3.7) after CMT vs. 10.4% (6.7–14.1) after ABVD alone (*p* = 0.0002, Fig. 2B), while out-field recurrences were more balanced between the groups (5-year cumulative incidence 3.7% [1.5–5.9] vs 6.4% [3.3–9.5], *p* = 0.37, data not shown). Most patients received high-dose chemotherapy (HDCT) and autologous stem-cell transplantation (ASCT) at progression or relapse (Table 1).

**Table 1:** Outcomes of the PET-2-negative per-protocol population.

	Standard CMT (*N* = 328)		ABVD alone (*N* = 300)	
Follow-up (months)				
For disease status	65 (64–67)		62 (58–64)	
For survival status	67 (65–72)		64 (62–69)	
Tumor events				
Progression	0		1	(<1%)
Early relapse (within one year after treatment)	2	(1%)	9	(3%)
Late relapse	14	(4%)	24	(8%)
Any tumor event	16	(5%)	34	(11%)
Second-line therapies				
HDCT and ASCT	8	(2%)	14	(5%)
DHAP or ICE without HDCT/ASCT	2	(1%)	0	
Other chemotherapy with or without radiotherapy	3	(1%)	7	(2%)
Radiotherapy only	1	(<1%)	7	(2%)
Antibody therapy	0		1	(<1%)
Relapse, but no second-line therapy	1	(<1%)	0	
Unknown second-line therapy	1	(<1%)	5	(2%)
Causes of death				
Hodgkin lymphoma	1	(<1%)	0	
Second primary malignancy^a^	4	(1%)	0	
Cardiovascular disease	1	(<1%)	1	(<1%)
Other disease (unspecified)	0		1	(<1%)
Accident	0		1	(<1%)
Unclear	3	(1%)	0	
Any event	9	(3%)	3	(1%)
Second primary malignancies				
Acute myeloid leukemia or myelodysplastic syndrome	0		1	(<1%)
Non-Hodgkin lymphoma	4	(1%)	1	(<1%)
Solid tumor	13	(4%)	10	(3%)
Any event	17	(5%)	12	(4%)

Data are median (95% CI) or *n* (%).

*CMT* combined-modality treatment, *ABVD* doxorubicin, bleomycin, vinblastine and dacarbazine, *HDCT* high-dose chemotherapy, *ASCT* autologous stem-cell transplantation, *DHAP* dexamethasone, cytarabine, and cisplatin, *ICE* ifosfamide, carboplatin, and etoposide.

^a^Pleural mesothelioma with hepatic filiae, urothelial carcinoma of the bladder with hepatic, osseous, lymphomatous, and adrenal filiae, progressive B-NHL, hepatocellular carcinoma.

SPM occurred in 17 and 12 patients treated with CMT and ABVD alone, respectively (Table 1), with corresponding 5-year cumulative incidences of 4.6% (2.1–7.1) vs. 4.2% (1.6–6.8, *p* = 0.57, Fig. 2C).

With a median follow-up for OS of 66 months, 9 and 3 patients have died, including four deaths from SPM and one from HL (Table 1). In the per-protocol analysis, estimated 5-year OS was 98.3% (96.9–99.8) with CMT and 98.8% (97.4–100) with ABVD alone (*p* = 0.14, Fig. 2D, ITT analysis: Supplementary Fig. 1B).

**Fig. 3:**
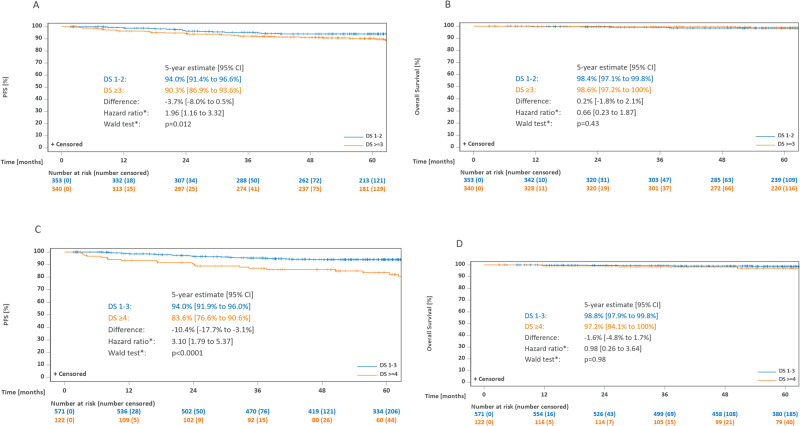
Kaplan-Meier estimates for PET-2-negative and PET-2-positive patients assigned to receive combined-modality treatment. **A** Progression-free survival, DS 1–2 vs DS ≥ 3. **B** Overall survival, DS 1–2 vs DS ≥ 3. **C** Progression-free survival, DS 1–3 vs DS ≥ 4. **D** Overall survival, DS 1–3 vs DS ≥ 4. Abbreviations: PET-2 positron emission tomography after two cycles of chemotherapy, PFS progression-free survival, DS, Deauville score. *Cox model adjusted for stratification factors age, sex, B symptoms, disease localization (supra- vs. infra diaphragmatic), albumin level (<4 g/dL vs. ≥4 g/dL), and bulky disease.

In addition to the 340 patients with a positive PET-2, i.e., DS ≥ 3, 353 patients with a negative PET-2, i.e., DS 1-2, were assigned to receive CMT. With a median follow-up of 64 months, estimated 5-year PFS was 94.0% (91.4–96.6) after a negative PET-2 and 90.3% (86.9–93.6) after a positive PET-2 (*p* = 0.012, Fig. 3A). With 9 and 8 deaths, respectively, OS was similar in the PET-2-negative and positive subgroups (98.4% [97.1–99.8] vs. 98.6% [97.2–100]; *p* = 0.43, Table 2, Fig. 3B). Using the less conservative and more commonly applied cut-off of DS 4 for PET-2 positivity, the prognostic value of PET-2 became more apparent with estimated 5-year PFS rates of 94.0% (91.9–96.0) after a DS 1–3 and 83.6% (76.6–90.6) after a DS ≥ 4 (*p* < 0.0001, Fig. 3C). Again, there was no OS difference (Fig. 3D). There were both more in-field and out-field recurrences in patients with positive PET-2 (Supplementary Fig. 3).

**Table 2:** Outcomes of PET-2-negative and PET-2-positive patients assigned to receive combined-modality treatment.

	PET-2-negative		PET-2-positive			
	DS 1–2 (*N* = 353)		DS 3 (*N* = 218)		DS ≥ 4 (*N* = 122)	
Follow-up (months)						
For disease status	65 (63–67)		63 (62–66)		64 (60–68)	
For survival status	67 (65–72)		65 (63–71)		65 (62–70)	
Tumor events						
Progression	0		0		6	(5%)
Early relapse (within one year after end of treatment)	3	(1%)	4	(2%)	2	(2%)
Late relapse	15	(4%)	12	(6%)	12	(10%)
Any tumor event	18	(5%)	16	(7%)	20	(16%)
Causes of death						
Hodgkin lymphoma	1	(<1%)			1	(1%)
Toxicity of second-line therapy	0		1	(<1%)	0	
Second primary malignancy	4	(1%)	2	(1%)	1	(1%)
Cardiovascular disease	1	(<1%)	1	(<1%)		
Unclear	3	(1%)	1	(<1%)	1	(1%)
Any event	9	(3%)	5	(2%)	3	(2%)

Data are median (95% CI) or *n* (%).

*PET-2* positron emission tomography after two cycles of chemotherapy, *DS* deauville score.

Cardiac toxicity after 5 years was not substantially different neither between treatment groups nor between male and female patients with regard to LVEF (Table 3).

**Table 3:** Cardiac toxicities.

	Standard CMT		PET-2-guided treatment		Total	
Female patients	*N*	Mean (SD)	*N*	Mean (SD)	*N*	Mean (SD)
LVEF (baseline), all available data	200	64.6% (8.3)	199	64.8% (7.8)	399	64.7% (8.0)
LVEF (baseline), only patients with follow-up data	37	66.7% (9.2)	35	64.4% (8.9)	72	65.6% (9.1)
LVEF (5-year follow-up)	37	63.4% (7.8)	35	65.1% (8.6)	72	64.2% (8.2)
Male patients	*N*	Mean (SD)	*N*	Mean (SD)	*N*	Mean (SD)
LVEF (baseline), all available data	279	63.8% (7.4)	264	63.6% (6.7)	543	63.7% (7.1)
LVEF (baseline), only patients with follow-up data	46	63.0% (9.7)	36	63.9% (5.2)	82	63.4% (8.0)
LVEF (5-year follow-up)	46	60.3% (8.7)	36	60.1% (6.5)	82	60.2% (7.7)

Data are mean (SD).

*CMT* combined-modality treatment, *LVEF* left ventricular ejection fraction.

**Fig. 4:**
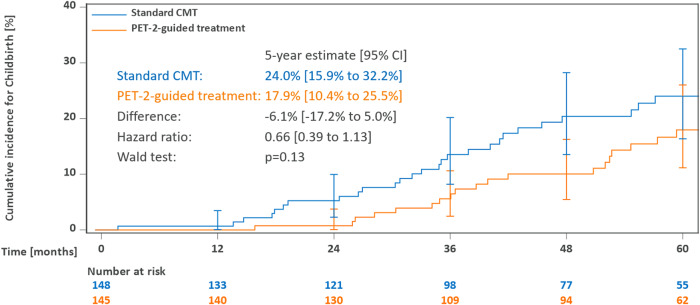
Cumulative incidence of first childbirth among female patients aged 18–40 years at enrollment. Abbreviations: CMT combined-modality treatment, PET-2 positron emission tomography after two cycles of chemotherapy.

Within a median follow-up of 63 months from the end of study therapy, 54 out of 293 women of age 18-40 at enrollment reported to have given birth. Cumulative incidences of childbirth after 5 years were 24.0% (15.9–32.2) in patients assigned to standard CMT and 17.9% (10.4–25.5) with PET-guided treatment (Fig. 4). Cryopreservation before start of therapy was documented in 10 (19%) of 54 women who later had children, and one reported to have used cryogenic material (fertilized oocytes, after receiving CMT as well as treatment for breast cancer).

Within a median follow-up of 62 months after therapy, 37 of 590 male patients aged 18–60 years at enrollment reported to have had children. In 28 (76%) of these patients, sperm cryopreservation had been done before therapy. However, information about whether cryogenic material had been used was not available.

## Discussion

This follow-up analysis of the GHSG HD16 trial confirms that RT cannot be omitted from the treatment of early-stage favorable HL after a negative PET-2 without a clinically relevant and statistically significant loss of efficacy. We could not observe any disadvantage of standard CMT over PET-2-guided treatment in terms of acute or late toxicities. OS remains on a high level and was - with the available follow-up - not impaired by the omission of RT, likely due to effective second-line therapies. We also could confirm PET-2-positivity as risk factor regarding PFS in patients receiving CMT, indicating the need for further improvement and innovative approaches in this group of patients.

In the primary HD16 analysis [13], non-inferiority of ABVD alone could be clearly ruled out in the primary per-protocol analysis. However, the sensitivity analysis in the ITT population showed a smaller PFS difference at that time. In the updated analysis presented herein, ITT results are much more pronounced and fully support the conclusion that omission of RT leads to inferior PFS in PET-2-negative patients.

The fact that in the CMT group of the ITT population only two relapses more have been observed compared with the per-protocol population, and both patients dropped out of the per-protocol population because they refused RT, supports this result as well. Moreover, the majority of relapses after ABVD alone were within a theoretical IF-RT field, highlighting an important role of RT in local tumor control and preventing in-field relapses.

Here, the question arises, how to improve the negative predictive value of PET-2 in order to allow for the omission of RT in selected patients. Novel tools such as circulating tumor DNA and metabolic tumor volume are currently under evaluation and might improve risk prediction in the future [16, 17].

Our results are in line with results from other randomized phase III trials such as the European Organisation for Research and Treatment of Cancer (EORTC)/The Lymphoma Study Association (LYSA)/Fondazione Italiana Linfomi (FIL) H10 and the UK National Cancer Research Institute (NCRI) RAPID studies [11, 18]. However, the authors of the RAPID trial drew different conclusions from their results by accepting a greater loss of efficacy for omitting RT, despite missing the pre-defined margin for non-inferiority. A joint analysis of the H10 and RAPID trials showed that relapse rates in patients with ABVD alone were higher than with CMT in the first two years. Afterwards, however, relapse rates in both groups were similar [19]. This assumption cannot be supported by our data and is in contrast to the findings in >20,000 early-stage HL patients from the US National Cancer Data Base, reporting superior OS with CMT versus chemotherapy only [20].

Even though PFS is significantly better in the standard CMT group compared to ABVD alone, OS continues to be very high in both groups – regardless of the higher rate of relapses for patients who did not receive RT. This can be attributed to the very effective second-line treatment options in HL. Most patients with relapse received HDCT and ASCT, which proves to be an effective treatment for relapse of HL, especially after initial early-stage disease [21–23]. Patients who received ABVD alone were treated with this intensive second-line therapy as well. Again, these results are in line with the results of H10 and RAPID [11, 18]. The authors of the RAPID trial concluded that the significant difference in PFS can be neglected since OS rates are still high in both groups. In line with the EORTC H10 investigators, we believe, however, that in spite of the high OS rates, the difference in PFS is clinically relevant and particularly of high importance for patients in terms of quality of life [24]. Additionally, while some patients were spared from first-line RT, they still received a very toxic and strenuous second-line treatment due to relapse to allow for these high OS rates. We postulate that most of these patients could have been spared from intensive second-line therapy if they would have had consolidation RT as part of CMT.

Of note, the findings from the present analysis comprising the whole study population of the HD16 trial and from the H10 and RAPID studies that only included individuals with classical HL also appear to hold true for patients with early-stage favorable nodular lymphocyte-predominant HL (NLPHL). A recent subgroup analysis of the patients with NLPHL treated within the HD16 study indicated that individuals presenting with this rare HL subtype also require consolidation RT irrespective of the PET-2 results to achieve the optimal disease control [25].

Importantly, there are not more SPM in the CMT group and therefore, late toxicities caused by RT seem to play a much smaller role than initially predicted – limited by the fact that the follow-up still is rather short and that SPM might occur 20 to 30 years after CMT. This is of great importance since late toxicities caused by RT have contributed to the decline in OS of HL patients in the past [26]. An analysis by Baues et al., including patients treated within the HD16 study, demonstrated that the rate of acute side effects with IF-RT was very low, with almost no grade 3 and mostly grade 1 toxicity [26]. This can be attributed to smaller irradiation fields, both due to technical improvements but also rather localized disease in early-stage HL given the absence of the risk factor ≥3 nodal areas and lower radiation doses. With the current standard consisting of involved-site RT, side effects are expected to decrease further [26]. Moreover, our data do neither demonstrate any strong negative impact on cardiac function measured by LVEF nor on fertility measured by the rate of child births.

With prolonged follow-up, PFS rates continue to be significantly higher for PET-2-negative patients. Even years after end of CMT, there are still more recurrences in patients with a positive PET-2, which are mostly located outside of the radiation field. Thus, improving response rates and importantly PFS in these patients could be achieved by intensifying chemotherapy instead of intensifying or modifying RT. This approach has been investigated in the EORTC H10 trial and showed promising results when intensifying chemotherapy to two cycles of escalated BEACOPP (bleomycin, etoposide, doxorubicin, cyclophosphamide, vincristine, procarbazine, prednisone) in case of PET-positivity after two cycles of ABVD [18].

Some of the inherent limitations of HD16 have been addressed already in the initial publication [13]. Additionally, although median follow-up is now 64 months, some late toxicities, such as SPMs or cardiovascular disease, may still not be evaluable sufficiently due to their delayed onset. In addition, data on birth rates and gonadal dysfunction, including whether or not in vitro fertilization was performed, is limited to a subset of patients due to limited data availability. Despite these limitations, the present update of this large international randomized phase III trial provides important long-term data and more robust findings than initially published.

In conclusion, this follow-up analysis of the GHSG HD16 trial confirms that RT cannot be omitted from treatment of early-stage favorable HL in case of a negative PET-2 without a significant and clinically relevant loss in efficacy. Accordingly, CMT remains standard treatment for early-stage HL patients. Since PET-2 positivity (≥DS 4) has a relevant negative impact on PFS, innovative treatment strategies are needed to improve outcomes after first-line treatment in these patients.

### Supplementary information

Supplemental Data
