# Supplementary material for: Follow-up of the GHSG HD16 trial of PET-guided treatment in early-stage favorable Hodgkin lymphoma
Source: Leukemia. 2023 Oct 16;38(1):160–7. doi: 10.1038/s41375-023-02064-y (PMC10776396; doi:10.1038/s41375-023-02064-y)
Supplement: Supplementary file 1 — Supplemental Data [file 41375_2023_2064_MOESM1_ESM.docx]

**Supplemental Data**

**Complete inclusion and exclusion criteria**

Inclusion criteria:

- Histologically proven primary diagnosis of Hodgkin lymphoma
- Classical Hodgkin lymphoma of clinical stage I or II or nodular lymphocyte-predominant Hodgkin lymphoma of Ann Arbor stage IB, IIA or IIB without any of the risk factors large mediastinal mass (≥ a third of the maximum transverse diameter of the thorax), extranodal lesions, elevated erythrocyte sedimentation rate (≥50 mm/h without B symptoms, ≥30 mm/h with B symptoms), or three or more involved lymph node areas.
- No previous treatment for Hodgkin lymphoma
- Age 18–75 years at inclusion
- Written informed consent to participate in the trial
- Consent to storage of data and tissue samples
- Normal organ function (except for Hodgkin lymphoma-related impairments)
- Life expectancy >3 months

Exclusion criteria:

- Incomplete diagnosis of the disease stage
- Prior or concurrent disease that prevents treatment according to protocol (please refer to trial protocol for details)
- Hodgkin lymphoma as part of a composite lymphoma
- Prior chemotherapy or radiation
- Malignant disease within the last five years (exceptions: basal cell carcinoma, carcinoma in situ of the cervix uteri, completely resected melanoma TNMpT1)
- Pregnancy, lactation
- Eastern Cooperative Oncology Group (ECOG) performance status >2
- Long-term ingestion of corticosteroids (e.g. for chronic polyarthritis) or antineoplastic drugs (e.g. methotrexate)
- Patient’s lack of accountability, inability to appreciate the nature, meaning and consequences of the trial and to formulate his/her own wishes correspondingly
- Noncompliance: Refusal of blood products during treatment, epilepsy, drug dependency, change of residence to abroad, prior cerebral injury, or similar circumstances that appear to make protocol treatment or long-term follow-up impossible
- Antiepileptic treatment
- General intolerance of any protocol medication
- Unsafe contraceptive methods (please refer to trial protocol for details)
- Relationship of dependence or employer-employee relationship to the sponsor or the investigator
- Commitment to an institution on judicial or official order
- Participation in another interventional trial that could interact with this trial.

**Chemotherapy**

ABVD was administered according to the following table:

| **Drug** | **Dose** | **Application** | **Day of cycle** |
| --- | --- | --- | --- |
| Doxorubicin | 25 mg/m² | IV | 1 and 15 |
| Bleomycin | 10 mg/m² | IV | 1 and 15 |
| Vinblastine | 6 mg/m² | IV | 1 and 15 |
| Dacarbazine | 375 mg/m² | IV | 1 and 15 |
| **ABVD regimen.** | | | |

Individual cycles were repeated on day 29.

Chemotherapy was generally administered in an out-patient setting. Blood counts were monitored weekly and additional liver and kidney function tests were performed once per cycle. Start of the next cycle was postponed until recovery if the white blood cells were less than 2500/µL or the platelet count less than 80000/µL on the day scheduled for retreatment or in case of serious unexpected non-hematological side-effects of Common Terminology Criteria for Adverse Events (CTCAE) grade 3 or 4. Treatment postponements of less than 15 days caused by inadequate blood value recovery only were no reason for dose reduction. In case of treatment postponements of 15 days or more, all drugs except for bleomycin should be reduced to 75%. Granulocyte colony-stimulating factor was given if clinically indicated.

**Radiotherapy**

Involved-field radiotherapy was centrally planned based on initial staging imaging; initial staging was revised if necessary. The recommended interval between completion of chemotherapy and starting radiotherapy was 4–6 weeks. The total dose of 20 Gy was given in fractions of 1.8–2.0 Gy five times per week.

**Staging examinations**

Staging and pre-treatment evaluation included medical history, physical examination, chest radiography, computed tomography (CT) scan of neck, chest, abdomen, and pelvis, ultrasound of the abdomen, biopsy of an involved lymph node or of another primarily involved organ, bone marrow biopsy, skeletal scintigraphy, serum chemistry, pulmonary function test, thyroid function test, electro- and echocardiography, and gonadal function test. Biopsy material was centrally reviewed by at least one member of a panel of six lymphoma pathology experts.

**Response assessment**

Response assessments were scheduled after two cycles of chemotherapy and, if applicable, four to six weeks after completion of radiotherapy. Response had to be documented for each initially involved region using adequate methods (CT scan, ultrasound, bone scintigraphy, bone marrow biopsy, bone marrow cytology). In doubtful cases, histological clarification was recommended. Additionally, ^18^F-FDG-PET was a mandatory examination after two cycles of chemotherapy (PET-2) for all patients with responding disease. PET-2 was performed between day 22 and day 35 of the second ABVD cycle. A multidisciplinary panel of experts from medical oncology, nuclear medicine, radiation oncology, and radiology centrally reviewed all imaging at staging and after two cycles ABVD and clinical information. The panel was masked to treatment group allocation and local findings and included some of the authors (MF, CK, GK, HE, CB, MD, PB, and AE). PET-2 was rated according to the Deauville score (DS) using the mediastinal blood pool as cut-off for PET positivity (DS 3 or higher). Patients with progressive disease were taken off study treatment.

During the follow-up period, physical examination, laboratory tests, chest X-ray, pulmonary function, abdominal ultrasound, thyroid diagnostics, electro- and echocardiography, assessment of gonadal function and self-assessment of quality-of-life were requested at defined intervals. Provided complete response had been reached, CT scans were to be performed only in cases of suspected tumor recurrence.

**Response criteria**

- Complete remission (CR): Disappearance of all clinical and radiological symptoms. Residual radiological abnormalities are compatible with CR in the definitive restaging in absence of signs of active lymphoma.
- Partial remission (PR): Continuing presence of lymphoma tissue (clinical, radiological) with significant reduction in all involved sites, shrinkage of the majority (at least half) of large involved lymph nodes and localized measurable organ manifestations (with more than 3 cm in diameter) by more than 50% compared with the initial status. If no lesion had more than 3 cm in diameter: shrinkage of the two largest involved lesions by more than 50% in greatest diameter. Shrinkage of a large mediastinal mass (if present) by more than 50% in the maximum transverse diameter, and absence of B symptoms and of rising or strongly elevated erythrocyte sedimentation rate.
- No change (NC): Active lymph node tissue present and criteria for PR not reached (ie, either overall too little shrinkage in all sites or inadequate shrinkage in particular sites or strongly elevated erythrocyte sedimentation rate and/or B symptoms) and no lesion enlarged by more than 25% in largest diameter.
- Progressive disease (PD): Occurrence of new lesions or increase of at least one already known lesion by more than 25% during or within three months after therapy.

**Statistical Analysis**

The study had two independent objectives. The primary objective was to show non-inferiority of treatment with ABVD alone as compared with standard combined-modality therapy (CMT) in terms of progression-free survival (PFS) among PET-2-negative patients. Clinically relevant inferiority was defined as a hazard ratio (HR) of 3.01 or more, based on an absolute difference of 10% in the 5-year PFS rates while assuming a 5-year PFS of 94.6% in the PET-2-negative CMT group. Non-inferiority would be established if the upper limit of the two-sided 95% CI for the HR was below 3.01. Assuming an actual inferiority of two percentage points of ABVD alone, the analysis could be performed with at least 80% power when at least 52 events for the primary endpoint PFS were observed in the per-protocol population of PET-2-negative patients. To this end, a total of 1150 patients had to be included in the trial, assuming that about 66% of patients would have a negative PET-2 and 10–15% would drop out of the per-protocol population.

The second objective of the study was to assess the prognostic impact of PET-2 among patients assigned to CMT. With the given trial design, a difference of at least 5% in 5-year PFS between PET-2-negative and PET-2-positive patients would be detected with a power of 80% and a two-sided significance level of 5%. Only patients with a valid PET-2 result assigned to receive CMT were to be included in the comparison, i.e. PET-2-positive patients from both arms and PET-2-negative patients from the CMT arm.

We compared time-to-event endpoints using the Kaplan-Meier method including HRs and 95% CIs obtained from univariate Cox regression models. To assess whether the prognostic impact of PET-2 is independent from baseline factors, sensitivity analyses for the comparison of PET-2-negative and PET-2-positive patients were performed including all stratification factors (except for center) in the regression model. Cumulative SMN incidence was estimated according to Kaplan-Meier accounting for death as a competing risk, and compared between treatment groups using subdistribution hazard ratios (sHRs) and 95% CIs obtained from Cox regression models. Other secondary endpoints were analyzed by means of descriptive statistics, with p values result from Fisher’s exact test where applicable. The non-inferiority test was primarily performed in the per-protocol population, excluding all patients with severe protocol deviations, as this was considered the most conservative analysis for non-inferiority objectives in the trial protocol. Sensitivity analyses and all other analyses were performed according to the intention-to-treat principle; however, all patients dropping out before central review of PET-2 had to be excluded from all analyses regarding the main objectives of the trial (ITT_PET_ population). We used SAS version 9.4 for all analyses. The trial was registered with ClinicalTrials.gov, number NCT00736320.

**Supplemental Tables**

| **Supplemental table 1.** Baseline characteristics of the intention-to-treat population | | | | | | |
| --- | --- | --- | --- | --- | --- | --- |
|  | **Combined-modality treatment (n=573)** | | **PET-2-guided treatment (n=566)** | | **Total**  **(n=1139)** | |
| **Age** |  | |  | |  | |
| Median (range) | 38 | (18–75) | 37 | (18–75) | 39 | (18–75) |
| 18–59 | 513 | (90%) | 506 | (89%) | 1019 | (89%) |
| 60–75 | 60 | (10%) | 60 | (11%) | 120 | (11%) |
| **Sex** |  |  |  |  |  |  |
| Female | 241 | (42%) | 244 | (43%) | 485 | (43%) |
| Male | 332 | (58%) | 322 | (57%) | 654 | (57%) |
| **Ann Arbor stage** |  |  |  |  |  |  |
| IA | 164 | (29%) | 145 | (26%) | 309 | (27%) |
| IB | 24 | (4%) | 22 | (4%) | 46 | (4%) |
| IIA | 358 | (62%) | 367 | (65%) | 725 | (64%) |
| IIB | 27 | (5%) | 32 | (6%) | 59 | (5%) |
| **ECOG performance status** |  |  |  |  |  |  |
| 0 | 529 | (92%) | 519 | (92%) | 1048 | (92%) |
| 1 | 43 | (8%) | 47 | (8%) | 90 | (8%) |
| 2 | 1 | (<1%) | 0 |  | 1 | (<1%) |
| **Initial involvement** |  |  |  |  |  |  |
| Supraclavicular | 289 | (50%) | 281 | (50%) | 570 | (50%) |
| Infraclavicular | 99 | (17%) | 92 | (16%) | 191 | (17%) |
| Upper mediastinal | 200 | (35%) | 203 | (36%) | 403 | (35%) |
| Lower mediastinal | 39 | (7%) | 36 | (6%) | 75 | (7%) |
| Lung hilus | 34 | (6%) | 26 | (5%) | 60 | (5%) |
| Any of the above | 354 | (62%) | 363 | (64%) | 717 | (63%) |
| **Histologic subtype** |  |  |  |  |  |  |
| Classical Hodgkin lymphoma | 433/476 | (91%) | 409/463 | (88%) | 842/939 | (90%) |
| Nodular lymphocyte predominant Hodgkin lymphoma | 43/476 | (9%) | 54/463 | (12%) | 97/939 | (10%) |
| Data are n (%) or n/total (%), unless otherwise indicated. Abbreviations: PET-2, positron emission tomography after two cycles of chemotherapy; ECOG­, Eastern Cooperative Oncology Group. | | | | | | |

| **Supplemental table 2.** Baseline characteristics of the PET-2-negative per-protocol population | | | | | | |
| --- | --- | --- | --- | --- | --- | --- |
|  | **Combined-modality treatment (n=328)** | | **ABVD alone (n=300)** | | **Total**  **(n=628)** | |
| **Age** |  | |  | |  | |
| Median (range) | 39 | (18–75) | 39 | (18–75) | 39 | (18–75) |
| 18–59 | 294 | (90%) | 261 | (87%) | 555 | (88%) |
| 60–75 | 34 | (10%) | 39 | (13%) | 73 | (12%) |
| **Sex** |  |  |  |  |  |  |
| Female | 138 | (42%) | 132 | (44%) | 270 | (43%) |
| Male | 190 | (58%) | 168 | (56%) | 358 | (57%) |
| **Ann Arbor stage** |  |  |  |  |  |  |
| IA | 105 | (32%) | 94 | (31%) | 199 | (32%) |
| IB | 16 | (5%) | 16 | (5%) | 32 | (5%) |
| IIA | 191 | (58%) | 175 | (58%) | 366 | (58%) |
| IIB | 16 | (5%) | 15 | (5%) | 31 | (5%) |
| **ECOG performance status** |  |  |  |  |  |  |
| 0 | 307 | (94%) | 276 | (92%) | 583 | (93%) |
| 1 | 20 | (6%) | 24 | (8%) | 44 | (7%) |
| 2 | 1 | (<1%) | 0 |  | 1 | (<1%) |
| **Initial involvement** |  |  |  |  |  |  |
| Supraclavicular | 161 | (49%) | 151 | (50%) | 312 | (50%) |
| Infraclavicular | 52 | (16%) | 39 | (13%) | 91 | (14%) |
| Upper mediastinal | 106 | (32%) | 87 | (29%) | 193 | (31%) |
| Lower mediastinal | 12 | (4%) | 13 | (4%) | 25 | (4%) |
| Lung hilus | 13 | (4%) | 9 | (3%) | 22 | (4%) |
| Any of the above | 191 | (58%) | 180 | (60%) | 371 | (59%) |
| **Histologic subtype** |  |  |  |  |  |  |
| Classical Hodgkin lymphoma | 254/275 | (92%) | 227/251 | (90%) | 481/526 | (91%) |
| Nodular lymphocyte predominant Hodgkin lymphoma | 21/275 | (8%) | 24/251 | (10%) | 45/526 | (9%) |
| Data are n (%) or n/total (%), unless otherwise indicated. Abbreviations: PET-2, positron emission tomography after two cycles of chemotherapy; ABVD, doxorubicin, bleomycin, vinblastine and dacarbazine; ECOG­, Eastern Cooperative Oncology Group. | | | | | | |

**Supplemental Figures**

**Supplemental figure 1.** Kaplan-Meier estimates for the PET-2-negative ITT_PET_ population: (A) Progression-free survival, (B) Overall survival.

Abbreviations: PET-2, positron emission tomography after two cycles of chemotherapy; ITT_PET_, intention-to-treat population excluding all patients dropping out before central review of PET-2; PFS, progression-free survival; CMT, combined-modality treatment; ABVD, doxorubicin, bleomycin, vinblastine and dacarbazine.

**A**

**
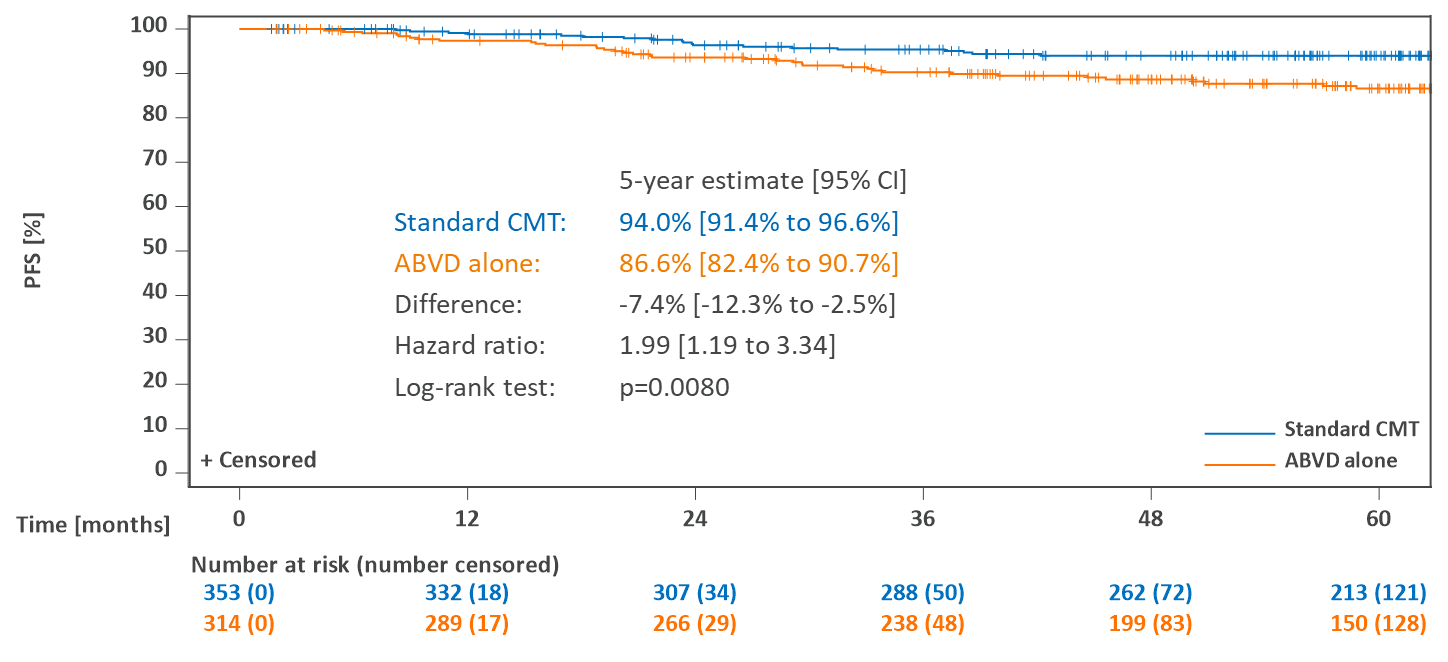
**

**B**

**
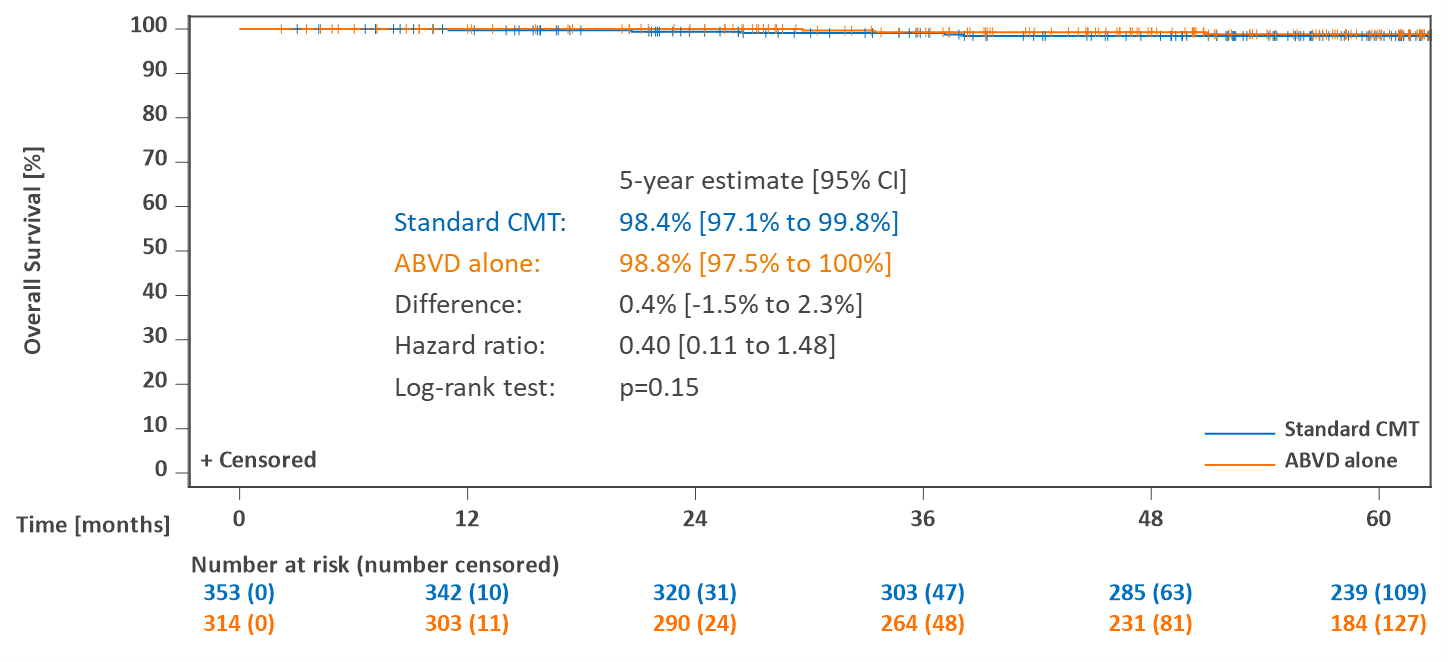
**

**Supplemental figure 2.** Kaplan-Meier estimates of progression-free survival for subgroups of the PET-2-negative per-protocol population: (A) Female patients, (B) Male patients, (C) Patients below the age of 50 years at enrollment.

Abbreviations: PET-2, positron emission tomography after two cycles of chemotherapy; PFS, progression-free survival; CMT, combined-modality treatment; ABVD, doxorubicin, bleomycin, vinblastine and dacarbazine.

**A**

**
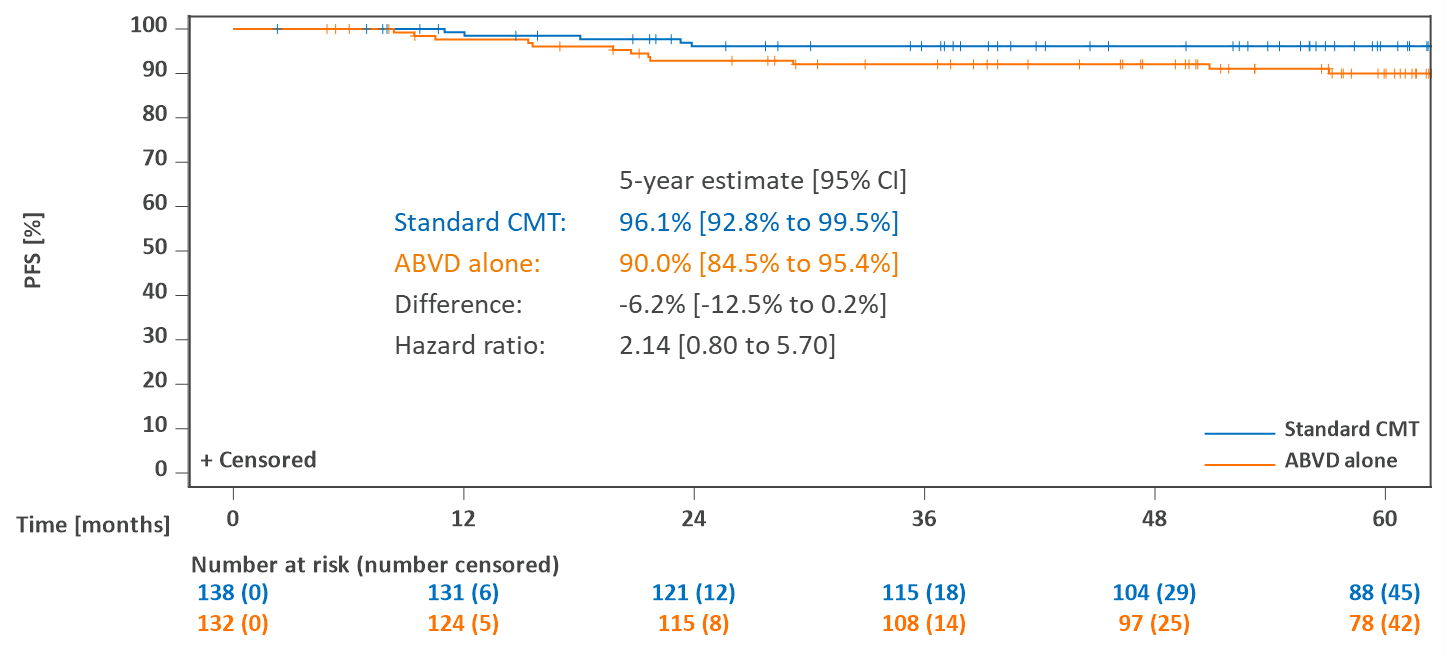
**

**B**

**
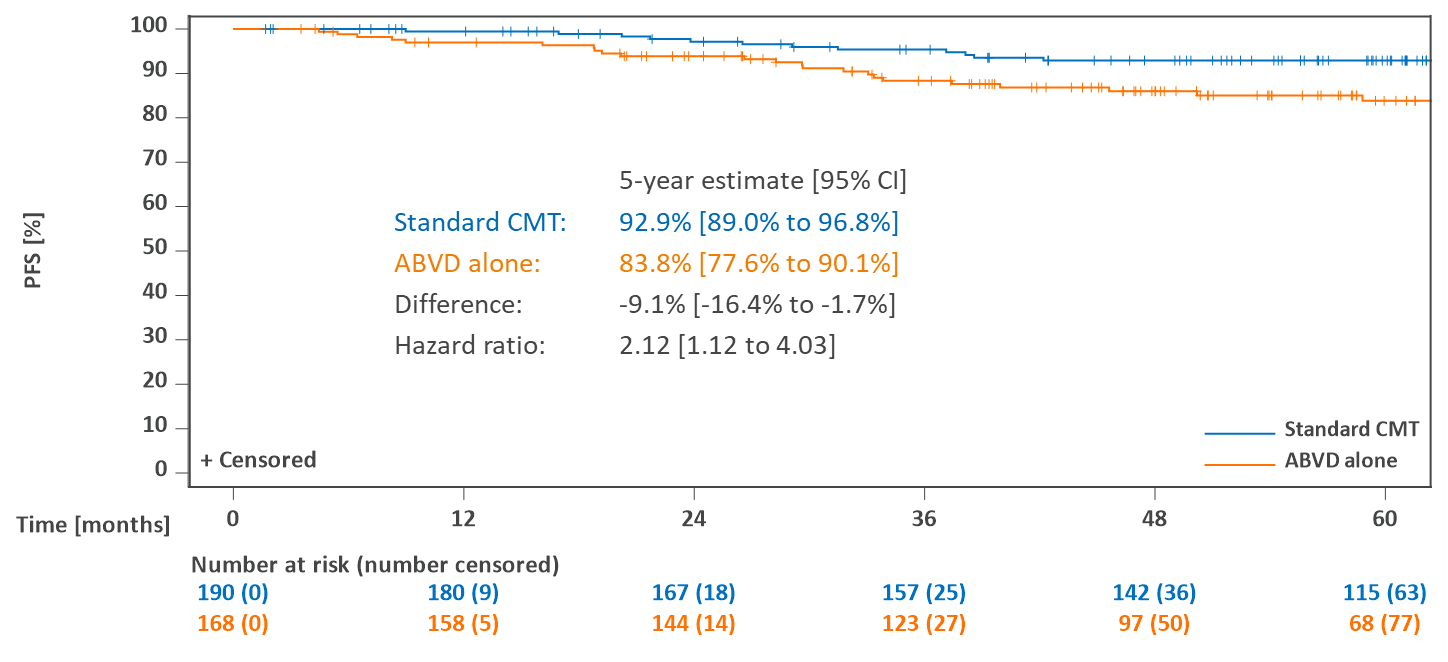
**

**C**

**
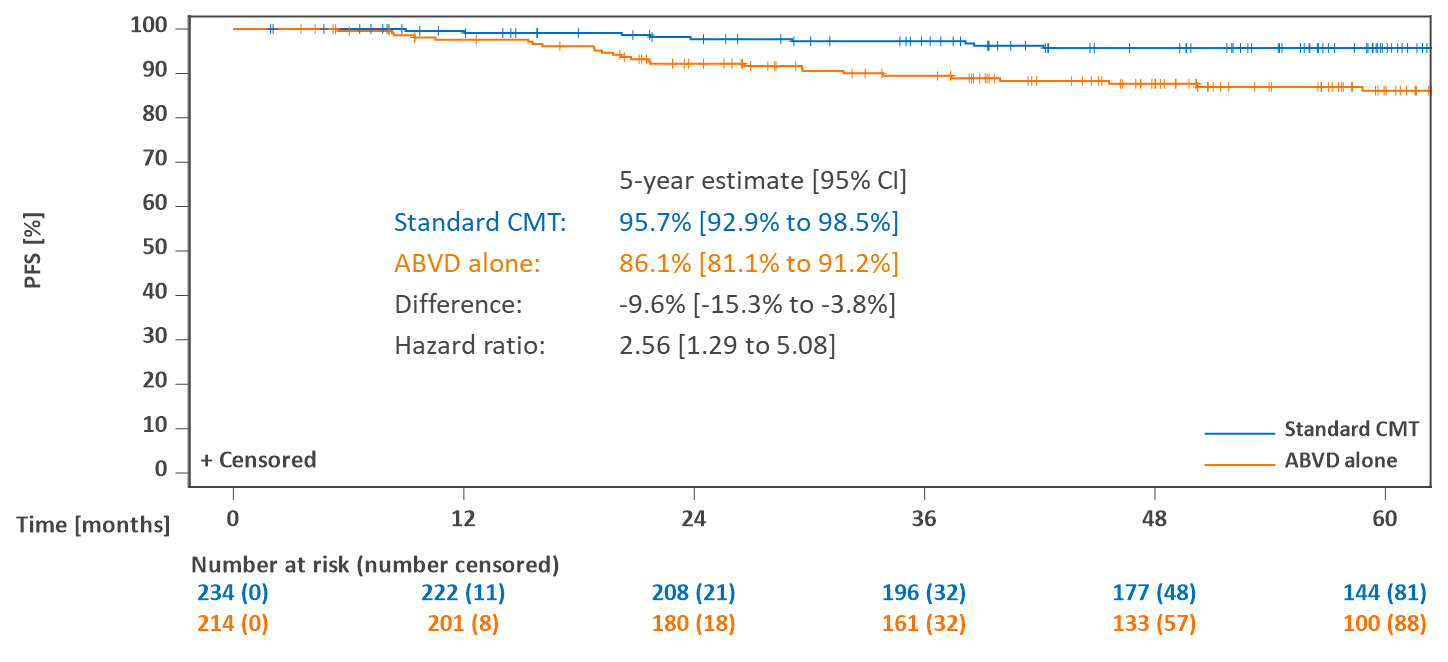
**

**Supplemental figure 3.** Cumulative incidence functions for in-field and out-field recurrences in PET-2-negative and PET-2-positive patients assigned to receive combined-modality treatment: (A) In-field recurrences, DS 1-2 vs DS ≥3, (B) Out-field recurrences, DS 1-2 vs DS ≥3, (C) In-field recurrences, DS 1-3 vs DS ≥4, (D) Out-field recurrences, DS 1-3 vs DS ≥4.

Abbreviations: PET-2, positron emission tomography after two cycles of chemotherapy; DS, Deauville score.

**A**

**
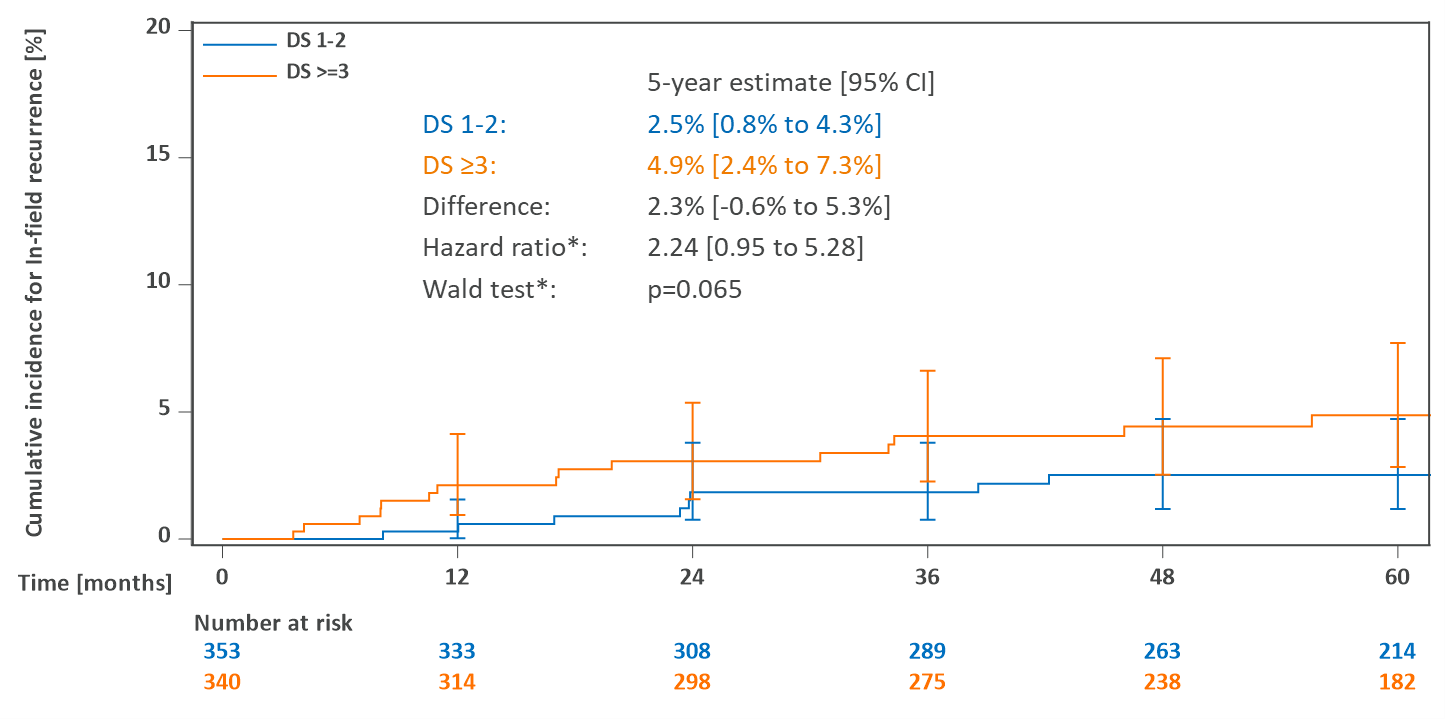
**

**B**

**
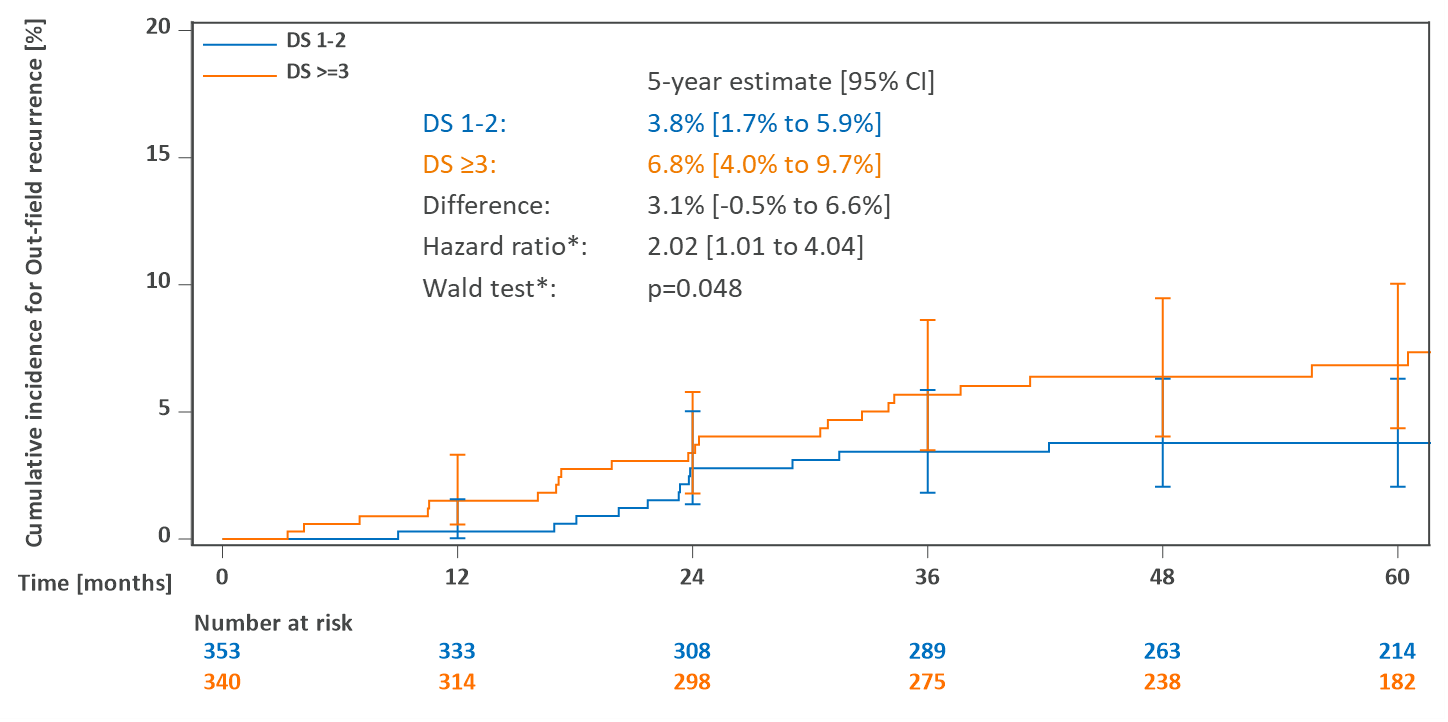
**

**C**

**
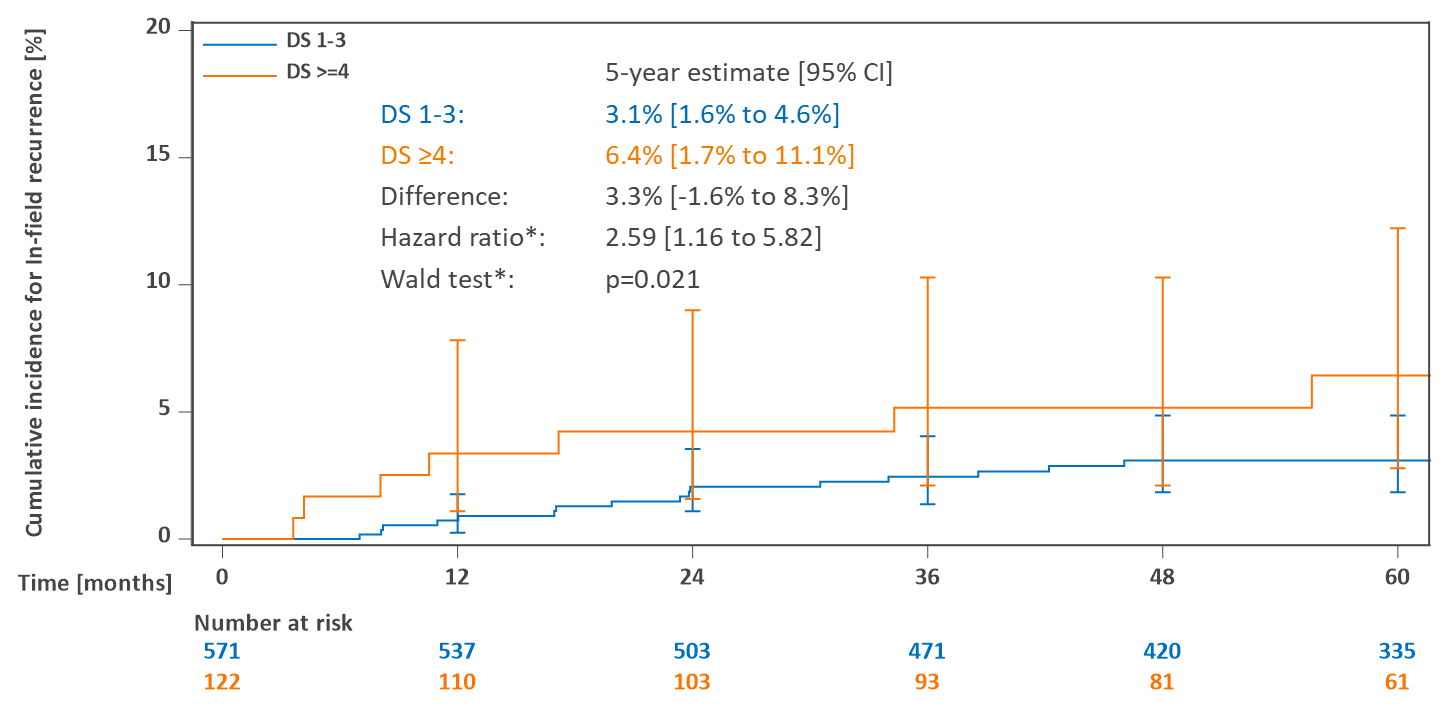
**

**D**

**
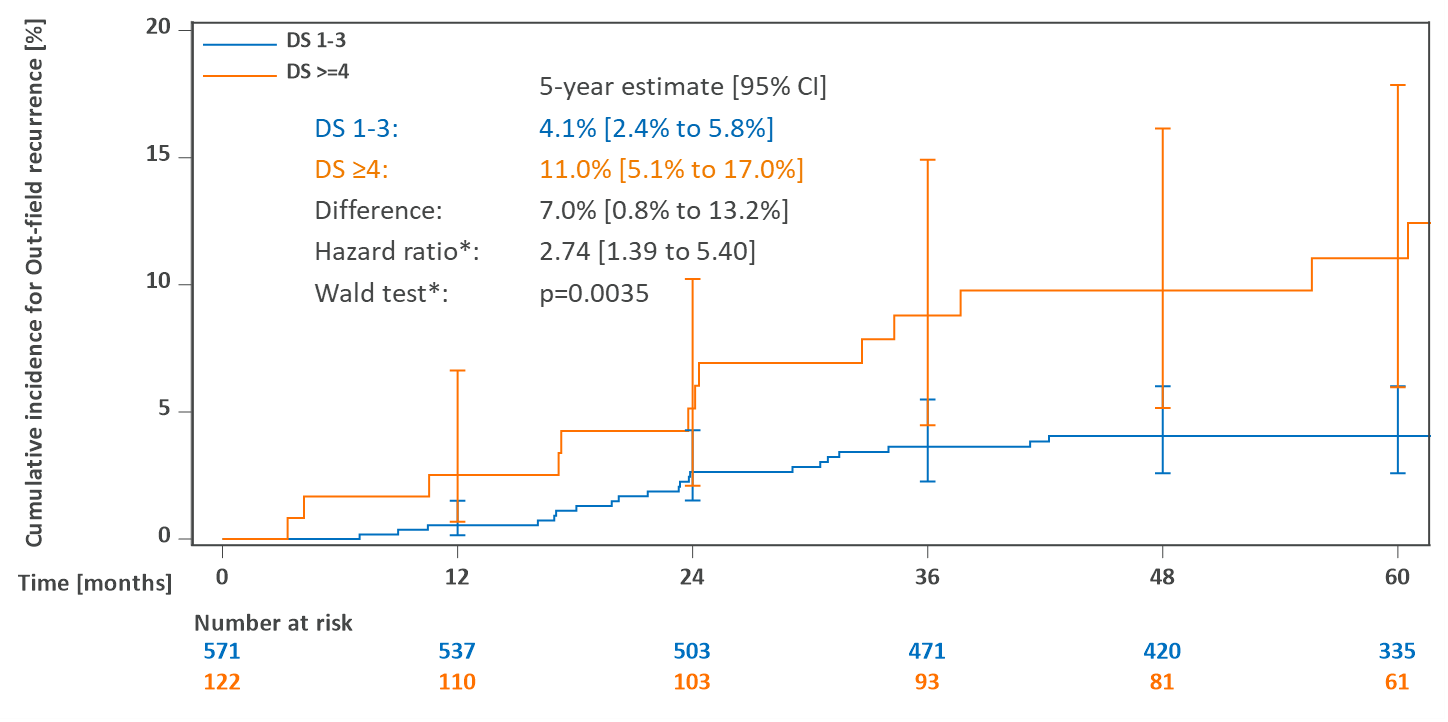
**

**Appendix: Participating trial sites**

| **Number of enrolled patients** | **Principal investigator, recruiting site (Germany unless indicated)** |
| --- | --- |
| 43 | Prof. Dr. med. Richard Greil, Innere Medizin III, AGMT gGmbH, Salzburg, Austria |
| 32 | Prof. Dr. med. Andreas Engert, Klinik I für Innere Medizin / Studienzentrum, Universitätsklinik Köln, Köln |
| 21 | Dr. med. Martin Vogelhuber, Klinik und Poliklinik für Innere Medizin III, Universitätsklinik Regensburg, Regensburg |
| 20 | Dr. med. Erhardt Schäfer, Studiengesellschaft Onkologie Bielefeld, Dres. med. Just/Düwel/Riesenberg/Steinke, Bielefeld |
| 20 | Prof. Dr. med. Max Topp, Med. Klinik und Poliklinik II, Universitätsklinikum Würzburg, Würzburg |
| 19 | Dr. med. J.M. Zijlstra-Baalbergen, Dept. of Hematology, VU University Medical Center, Amsterdam, The Netherlands |
| 19 | Dr. med. Martin Sökler, GCP-Studienzentrale der Inneren Medizin II, Eberhard-Karls-Universität, Tübingen |
| 18 | Prof. Dr. med. Bernd Hertenstein, Med. Klinik I, Abt. Hämatologie/ Onkologie, Klinikum Bremen Mitte gGmbH, Bremen |
| 17 | Prof. Dr. Ulrich Bernd Keller, Medizinische Klinik III, Klinikum "Rechts der Isar", München |
| 13 | Dr. med. Andrea Kerkhoff, Innere Medizin A, Universitätsklinik Münster, Münster |
| 12 | Prof. Dr. med. Stefan Krause, Medizinische Klinik V, Universitätsklinik Erlangen, Erlangen |
| 12 | Prof. Dr. med. Martin Wilhelm, Klinik V Onkologie / Hämatologie, Klinikum Nürnberg, Nürnberg |
| 12 | Dr. med. Andreas Viardot, Innere Abteilung III, Universitätsklinikum Ulm, Ulm |
| 12 | Prof. Dr. med. Georg Maschmeyer, Medizinische Klinik, Hämatologie/ Onkologie, Klinikum Ernst von Bergmann, Potsdam |
| 11 | Dr. med. Julia Meissner, Medizinische Klinik und Poliklinik V, Universitätsklinikum Heidelberg, Heidelberg |
| 11 | Dr. med. Julia Thiemer, Innere Medizin Hämatologie / Onkologie, Klinikum der Philipps-Universität, Marburg |
| 11 | Prof. Dr. med. Ulrich Dührsen, Klinik für Hämatologie / WTZ Ambulanz, Universitätsklinik Essen, Essen |
| 10 | Prof. Dr. med. Helga Bernhard, Med. Klinik V / Hämatologie, Klinikum Darmstadt GmbH, Darmstadt |
| 10 | Dr. med. Sonja Martin, Innere Medizin II, Hämatologie / Onkologie, Robert-Bosch-Krankenhaus, Stuttgart |
| 10 | Dr. med. Miriam Ahlborn, Medizinische Klinik III, Städtisches Klinikum Braunschweig, Braunschweig |
| 10 | Dr. med. Teresa Halbsguth, Innere Medizin, Hämatologie u. Onkologie (Hodgkin-Studien), Universitätsklinikum Frankfurt am Main, Frankfurt |
| 9 | Gudrun Schüler, Schwerpunktpraxis, Cottbus |
| 9 | Prof. Dr. med. Heinz-Gert Höffkes, Tumorklinik, MVZ Osthessen, Klinikum Fulda, Fulda |
| 9 | Priv. Doz. Dr. med. Thomas Pabst, Klinik und Poliklinik für Med. Onkologie, Inselspital Bern, Bern, Switzerland |
| 9 | Prof. Dr. med. Helmut Ostermann, Med. Klinik III für Hämatologie / Onkologie, Klinikum Großhadern, München |
| 9 | Dr. med. Joachim Haessner, Gemeinschaftspraxis, Wolfsburg |
| 9 | Priv. Doz. Dr. med. Bernd Metzner, Innere Medizin II, Klinikum Oldenburg, Oldenburg |
| 9 | Dr. med. Felicitas Hitz, Onkologie, Kantonsspital St. Gallen, St. Gallen, Switzerland |
| 8 | Prof. Dr. med. Stephan Mathas, Medizinische Klinik, Hämatologie / Onkologie, Campus Virchow Klinikum, Berlin |
| 8 | Dr. med. Christoph Plöger, Mannheimer Onkologiepraxis, Mannheim |
| 8 | Prof. Dr. med. Ullrich Graeven, Medizinische Klinik I, Kliniken Maria Hilf GmbH, Mönchengladbach |
| 8 | Prof. Dr. med. Dietger Niederwieser, Medizinische Klinik II, Hämatologie, Universitätsklinik Leipzig, Leipzig |
| 8 | Dr. med. Tobias Gaska, Klinik für Hämatologie / Onkologie, Brüderkrankenhaus St. Josef Paderborn, Paderborn |
| 8 | Dr. med. Christina Große-Thie, Medizinische Klinik III, Hämatologie, Onkologie, Palliativmedizin, Universitätsklinikum Rostock AöR, Rostock |
| 8 | Dr. med. Dagmar Kühnhardt, Zentrum für Innere Medizin, Hämatologie / Onkologie, Charité Campus Mitte, Berlin |
| 8 | Priv. Doz. Dr. med. Kathleen Jentsch-Ullrich, Gemeinschaftspraxis, Magdeburg |
| 8 | Dr. med. Walter Lindemann, Klinik für Hämatologie und Onkologie, Katholisches Krankenhaus Hagen GmbH, Hagen |
| 8 | Dr. med. Thomas Illmer, Gemeinschaftspraxis, Dresden |
| 8 | Dr. med. Stefan Fronhoffs, Onkologische Gemeinschaftspraxis, Siegburg |
| 7 | Prof. Dr. med. Uwe Martens, Medizinische Klinik III, SLK- Kliniken Heilbronn GmbH, Heilbronn |
| 7 | Dr. med. Heinz Dürk, Innere Abteilung, St. Marien Hospital Hamm, Hamm |
| 7 | Prof. Dr. med. Christian Meyer zum Büschenfelde, Medizinische Klinik, Abt. II Hämatologie / Onkologie, Vincentius-Diakonissen-Kliniken gAG, Karlsruhe |
| 7 | Dr. med. Georg Schliesser, Gemeinschaftspraxis Hämatologie-Onkologie Gießen, Gießen |
| 7 | Dr. med. Stefan Fuxius, Onkologische Schwerpunktpraxis Heidelberg, Heidelberg |
| 7 | Prof. Dr. med. Christian Könecke, Abteilung Hämatologie / Onkologie, Med. Hochschule Hannover, Hannover |
| 7 | Dr. med. Matthias Zaiss, Onkologische Schwerpunktpraxis, Freiburg |
| 7 | Prof. Dr. med. Yon Ko, Innere Medizin I, Johanniter-Krankenhaus, Bonn |
| 7 | Prof. Dr. med. Norbert Frickhofen, Innere Medizin III, Hämatologie / Onkologie, Wiesbaden |
| 7 | Prof. Dr. med. Alexander Kiani, Medizinische Klinik IV, Klinikum Bayreuth, Bayreuth |
| 7 | Dr. med. Hans Juergen Salwender, Medizinische Klinik II, Asklepios Klinik Altona, Hamburg |
| 7 | Dr. med. Georg Jacobs, Praxis für Hämatologie und Onkologie, Saarbrücken |
| 7 | Dr. med. Wolfram Jung, Abteilung Hämatologie und Onkologie, Universitätsklinikum der Georg-August-Universität, Göttingen |
| 6 | Dr. med. Harald Biersack, Hämatologie / Onkologie, Med. Klinik I, Universitätsklinikum Schleswig-Holstein, Lübeck |
| 6 | Dr. med. Karin Hohloch, med Oncology, Kantonsspital Graubünden, Chur, Switzerland |
| 6 | Dr. med. Jan Wierecky, Überörtliche Gemeinschaftspraxis, Hamburg |
| 6 | Dr. med. Thomas Geer, Innere Abteilung, Diakonie-Krankenhaus Schwäbisch Hall, Schwäbisch Hall |
| 6 | Dr. med. Andreas Rank, Medizinische Klinik II, Klinikum Augsburg, Augsburg |
| 6 | Dr. med. Hans-Joachim Beck, Abteilung für Hämatologie, III. Med. Klinik, Gebäude 605, Universitätsklinik Mainz, Mainz |
| 6 | Dr. med. Irmgard Dresel, Medizinische Klinik II, Hämatologie, Onkologie, Ortenau Klinikum Offenburg Gengenbach, Offenburg |
| 6 | Prof. Dr. med. Mathias Rummel, Med. Klinik IV, Hämatologie / Onkologie, Justus-Liebig-Universität Gießen, Gießen |
| 6 | Dr. med. Dennis Hahn, Klinik für Onkologie, Klinikum Stuttgart, Stuttgart |
| 6 | Mike Haberkorn, Hämatologische & Onkologische Tagesklinik Landshut, VK & K Studien GbR, Landshut |
| 6 | Prof. Dr. med. Judith Dierlamm, Onkologisches Zentrum, Abt. Hämatologie / Onkologie, Universitätsklinikum Hamburg-Eppendorf, Hamburg |
| 6 | Prof. Dr. med. Holger Hebart, Zentrum für Innere Medizin, Kliniken Ostalb, Mutlangen |
| 6 | Dr. med. Albert von Rohr, Onkozentrum, Klinik Hirslanden, Zürich, Switzerland |
| 6 | Dr. med. Joachim Zimber, Internistische Gemeinschaftspraxis, Nürnberg |
| 6 | Prof. Dr. med. Michael Pfreundschuh, Innere Medizin I, Universitätsklinikum des Saarlandes, Homburg |
| 6 | Dr. med. Siegfried Haas, Hämatologie, Onkologie u. Nephrologie, FEK Friedrich-Ebert-Khs.Neumünster GmbH, Neumünster |
| 6 | Dr. med. Johannes Mohm, Gemeinschaftspraxis, Dresden |
| 6 | Dr. med. Rolf Mahlberg, Medizinische Klinik, Krankenanstalt Mutterhaus d. Borromäerinnen, Trier |
| 5 | Dr. med. Kathrin Nachtkamp, Abteilung für Hämatologie, Onkologie, Universitätsklinikum Düsseldorf, Düsseldorf |
| 5 | Dr. med. Peter Moosmann, Zentrum für Onkologie/Hämatologie, Kantonsspital Aarau AG, Aarau, Switzerland |
| 5 | Prof. Dr. med. Volker Runde, Hämatologie/Onkologie, Wilhelm-Anton-Hospital gGmbH Goch, Goch |
| 5 | Dr. med. Roland Rudolph, Praxis Innere Medizin, Essen |
| 5 | Prof. Dr. med. Bertram Glaß, Hämatologie, Onkologie und Tumorimmunologie, HELIOS Klinikum Berlin-Buch, Berlin |
| 5 | Dipl.-Med. Steffen Dörfel, Onkologische Gemeinschaftspraxis, Dresden |
| 5 | Dr. med. Gerold Baake, Hämat.-Onkologische Schwerpunktpraxis, Pinneberg |
| 5 | Dr. med. Erik Engel, HOPA MVZ GmbH, , Hamburg |
| 5 | Dr. med. Fatime Krasniqi, Onkologie, Universitätsspital Basel, Basel, Switzerland |
| 5 | Dr. med. Christian Lerchenmüller, Praxis, Münster |
| 5 | Prof. Dr. med. Stephan Schmitz, Onkologische Schwerpunktpraxis, Köln |
| 5 | Dr. med. Oswald Burkhard, Internistische Gemeinschaftspraxis, Worms |
| 5 | Dr. med. Daniel Schöndube, Klinik für Hämatologie, Onkologie und Palliativmedizin, HELIOS Klinikum Bad Saarow, Bad Saarow |
| 5 | Dr. med. Elisabeth Lange, Medizinische Klinik, Hämatologie / Onkologie, Evangelisches Krankenhaus Hamm, Hamm |
| 5 | Dr. med. Oliver Schmah, Klinik für Hämatologie / Onkologie, Caritasklinik St. Theresia, Saarbrücken |
| 5 | Prof. Dr. med. Michael Kiehl, Klinik für Innere Medizin, Hämatologie / Internistische Onkologie, Klinikum Frankfurt/Oder GmbH, Frankfurt/ Oder |
| 5 | Prof. Dr. med. Frank Griesinger, Hämatologie und Internistische Onkologie, Pius Hospital, Oldenburg |
| 5 | Prof. Dr. med. Gerhard Held, Innere Medizin I / Hämatologie, Westpfalz Klinikum GmbH, Kaiserslautern |
| 5 | Dr. med. Rudolf Peceny, Hämatologie, Onkologie und Blutstammzelltransplantation, Klinikum Osnabrück GmbH, Osnabrück |
| 4 | Prof. Dr. med. Ulrich Kaiser, Medizinische Klinik II, St. Bernward-Krankenhaus, Hildesheim |
| 4 | Dr. med. Martin Klump, Medizinische Klinik, Ev. Diakonie Klinikum Jung-Stilling-Krankenhaus, Siegen |
| 4 | Prof. Dr. med. Rudolf Weide, InVO-Institut für Versorgungsforschung in der, Onkologie GbR, Koblenz |
| 4 | Dr. med. Sigrun Müller-Hagen, Dres. Müller-Hagen/Bertram, Forschungsgesell. für Malignität und Resultate GbR, Hamburg |
| 4 | Dr. med. Johannes Meiler, MVZ, Stade |
| 4 | Prof. Dr. med. Thomas Decker, Onkologie Ravensburg, Ravensburg |
| 4 | Prof. Dr. med. Gerhard Heil, Innere Abteilung / Onkologie, Klinikum Lüdenscheid, Lüdenscheid |
| 4 | Priv. Doz. Dr. med. Emanuele Zucca, Oncology, Istituto Oncologico della Svizzera Italiana, Bellinzona, Switzerland |
| 4 | Prof. Dr. med. Michael Heike, Medizinische Klinik / Onkologie, Kliniken Dortmund gGmbH, Dortmund |
| 4 | Dr. med. Mohammed Wattad, Zentrum für Innere Medizin, Evangelisches Krankenhaus, Essen |
| 4 | Dr. med. Burkhard Schmidt, Onkologische Schwerpunktpraxis, München |
| 4 | Dr. med. Manfred Glados, Onkologische Schwerpunktpraxis, Coesfeld |
| 4 | Dr. med. Christoph Diekmann, Med. Klinik Abt. Hämatologie / Onkologie, Evang. Diakoniekrankenhaus, Bremen |
| 4 | Prof. Dr. med. Martin Bentz, Medizinische Klinik III, Städtisches Klinikum Karlsruhe, Karlsruhe |
| 4 | Dr. med. Lars-Olof Mügge, Klinik für Innere Medizin III + MVZ Hämatologie/Onkologie 2, Heinrich-Braun-Klinikum Zwickau gGmbH, Zwickau |
| 4 | Priv. Doz. Dr. med. Jan Schröder, Gemeinschaftspraxis, Mülheim |
| 4 | Dr. med. Hans Werner Tessen, MVZ Onkologische Kooperation Harz, , Goslar |
| 4 | Priv. Doz. Dr. med. Gerald Meckenstock, Abteilung für Onkologie und Hämatologie, St. Josef-Hospital Gelsenkirchen-Horst, Gelsenkirchen |
| 4 | Dr. med. Christian Marin, Abteilung Hämatologie / Onkologie, Helios Kliniken Schwerin, Schwerin |
| 4 | Dr. med. Marcus Grüner, Gemeinschaftspraxis, Weiden |
| 4 | Priv. Doz. Dr. med. Stefan Balabanov, Onkologie, Universitätsspital Zürich, Zürich, Switzerland |
| 4 | Prof. Dr. med. Markus Bangerter, Gemeinschaftspraxis Innere Medizin, Augsburg |
| 4 | Dr. med. Karin Heinisch, Medizinische Klinik II, Klinikum Lippe-Lemgo, Lemgo |
| 4 | Dr. med. Jürgen Gatter, Innere Medizin III - Hämatologie und Onkologie, Klinikum Kempten / Oberallgäu gGmbH, Kempten / Allgäu |
| 4 | Dr. med. Reinhard Depenbusch, Onkodok GmbH, Gütersloh |
| 4 | Dr. med. Gerald Gehbauer, MVZ Onkologie Ingolstadt, Ingolstadt |
| 4 | Dr. med. Heike Schieder, Praxis, Buchholz |
| 4 | Dr. med. Stephan Kremers, Praxis für Innere Medizin und Hämatologie, Caritas-Krankenhaus, Lebach |
| 4 | Dr. med. Marion Schmalfeld, Gemeinschaftspraxis für Innere Medizin, Halle |
| 4 | Dr. med. Claudia Baum, Klinik für Hämatologie und Onkologie, Marienhospital, Düsseldorf |
| 4 | Prof. Dr. med. Tim H. Brümmendorf, Medizinische Klinik IV, Med. Fakultät der RWTH Aachen, Aachen |
| 3 | Prof. Dr. med. Michael Koenigsmann, Ges. für Medizinstatistik + Projektentwicklung, Hannover |
| 3 | Dr. med. Peter Schmidt, Praxis, Neunkirchen |
| 3 | Dr. med. Johannes Spes, Innere Medizin, Kreisklinik Altötting, Altötting |
| 3 | Dr. med. Georg Köchling, Netzwerk Onkologie, Schwarzwald-Baar-Heuberg - Standort Villingen, Villingen-Schwenningen |
| 3 | Dr. med. Johannes Schneider, Innere Medizin I, Klinikum Idar-Oberstein GmbH, Idar-Oberstein |
| 3 | Dr. med. Volkmar Böhme, OncoResearch Lerchenfeld GmbH, Hamburg |
| 3 | Priv. Doz. Dr. med. Mathias Hänel, Klinik für Innere Medizin III / Studiensekretariat, Klinikum Chemnitz, Chemnitz |
| 3 | Dr. med. Burkhard Otremba, Praxisgemeinschaft, Oldenburg |
| 3 | Prof. Dr. med. Sebastian Fetscher, Abteilung Hämatologie / Onkologie, Sana Kliniken Lübeck GmbH, Lübeck |
| 3 | Dr. med. Heike Knipp, Innere Medizin I, Alfried-Krupp-Krankenhaus, Essen |
| 3 | Dr. med. Oliver Schmalz, Med. Klinik I, Hämatologie/ Onkologie, Helios Klinikum Wuppertal, Wuppertal |
| 3 | Dr. med. Ludwig Fischer von Weikersthal, Onkologie/Hämatologie, Gesundheitszentrum St. Marien GmbH, Amberg |
| 3 | Dr. med. Birgit Schmid, Innere Medizin III, Marienhospital, Stuttgart |
| 3 | Dr. med. Sebastian Müller, Gemeinschaftspraxis Innere Medizin, Ansbach |
| 3 | Priv. Doz. Dr. med. Christian Scholz, Klinik für Innere Medizin, Vivantes Klinikum am Urban, Berlin |
| 3 | Dr. med. Ute Kreiter, Internistische Schwerpunktpraxis, Mainz |
| 3 | Prof. Dr. med. Dirk Behringer, Klinik für Hämatologie, Onkologie und Palliativmedizin, Augusta-Kranken-Anstalt gGmbH, Bochum |
| 3 | Sven Dyrda, Klinik für Internistische Onkologie / Hämatologie, Kliniken Essen-Mitte, Essen |
| 3 | Dr. med. Markus Sieber, Medizinische Klinik, Kreiskrankenhaus Gummersbach, Gummersbach |
| 3 | Dr. med. Thomas Zehrfeld, Innere Medizin Hämatologie/Onkologie, Kreiskrankenhaus Torgau, Torgau |
| 3 | Dr. med. Martin Hoffmann, Medizinische Klinik A, Klinikum der Stadt Ludwigshafen, Ludwigshafen |
| 3 | Prof. Dr. med. Daniel Betticher, Innere Medizin, Kantonsspital Friburg, Fribourg, Switzerland |
| 3 | Dr. med. Martine Klausmann, Gemeinschaftspraxis, Aschaffenburg |
| 3 | Natalie Fischer, Medizinische Onkologie, Kantonsspital Winterthur, Winterthur, Switzerland |
| 3 | Dr. med. Matthias Respondek, Praxis für Innere Medizin, Stuttgart |
| 3 | Dr. med. Christoph Schimmelpfennig, Klinik für Internistische Onkologie und Hämatologie, Klinikum St. Georg gGmbH, Leipzig |
| 3 | Dr. med. Georgi Kojouharoff, Onkologische Schwerpunktpraxis Darmstadt, , Darmstadt |
| 3 | Priv. Doz. Dr. med. Michael Flaßhove, Medizinische Klinik III, Krankenhaus Düren gGmbH, Düren |
| 3 | Dr. med. Oliver Stötzer, Hämatologische Schwerpunktpraxis, München |
| 3 | Prof. Dr. med. Dirk Strumberg, Abteilung Hämatologie / Onkologie, Marienhospital Herne, Herne |
| 2 | Dr. med. Ali Aldaoud, Praxis Dr. med. Ali Aldaoud, Leipzig |
| 2 | Dr. med. Doris Fleckenstein, MVZ MOP Elisenhof, , München |
| 2 | Priv. Doz. Dr. med. Christoph Kahl, gemeinnützige GmbH, Innere Medizin Abt. Hämatologie / Onkologie, Klinikum Magdeburg, Magdeburg |
| 2 | Priv. Doz. Dr. med. Peter Staib, Abt. für Hämatologie / Onkologie, St. Antonius Hospital, Eschweiler |
| 2 | Dr. med. Freerk Müller, Praxis, Verden |
| 2 | Dr. med. Alexander Wacker, Med. Klinik I, Kreiskliniken Reutlingen GmbH, Reutlingen |
| 2 | Dr. med. Beate Dargel, Med. Klinik, Abt. Hämato-/Onkologie in Koop. mit MVZ-Praxis, Harzklinikum Dorothea Christiane Erxleben GmbH, Wernigerode |
| 2 | Dr. med. Gerhard Puchtler, Medizinische Klinik II, Onkologische Tagesklinik, RoMed Klinikum, Rosenheim |
| 2 | Dr. med. Florian Fauth, Gemeinschaftspraxis, Hanau |
| 2 | Dr. med. Daniel Rauch, Med.-Onkologie, SpitalSTS AG Simmenthal-Thun-Saanenland, Thun, Switzerland |
| 2 | Dr. med. Cornelia Winkelmann, Innere Medizin, Evangelisches Krankenhaus, Lutherstadt Wittenberg |
| 2 | Dr. med. Monika Ebnöther, Onkologie, St. Claraspital Basel, Basel, Switzerland |
| 2 | Dr. med. Dietmar Reichert, Gemeinschaftspraxis für Innere Medizin, Westerstede |
| 2 | Prof. Dr. med. Thomas Neuhaus, Abt. Hämatologie, St. Vincenz Krankenhaus, Limburg |
| 2 | Dr. med. Lothar Müller, Schwerpunktpraxis, Leer |
| 2 | Dr. med. Alexander Regnery, Onkologisches Zentrum, AMEOS Klinikum, Bremerhaven |
| 2 | Priv. Doz. Dr. med. Michael Sandherr, Gemeinschaftspraxis, Weilheim |
| 2 | Dr. med. Yves Dencausse, Innere Medizin, Siloah St. Trudpert Klinikum, Pforzheim |
| 2 | Dr. med. Hans-Peter Laubenstein, Gemeinschaftspraxis Innere Medizin - DokuMed, Trier |
| 2 | Dr. med. Margarete Plath, Gemeinschaftspraxis, Augsburg |
| 2 | Prof. Dr. med. Rudolf Pihusch, Praxis Pihusch, Medizinisches Versorgungszentrum GbR, Rosenheim |
| 2 | Dr. med. Thomas Kubin, Med.Abteilung 5/3 Hämatologie, Klinikum Traunstein, Traunstein |
| 2 | Dr. med. Dennis Hahn, Medizinische Klinik I, Bürgerhospital Stuttgart, Stuttgart |
| 2 | Dr. med. Mathias Schmid, Institut für med. Onkologie und Hämatologie, Stadtspital Triemli, Zürich, Switzerland |
| 2 | Dr. med. Michael Schaefers, Gemeinschaftspraxis Innere Medizin, Duisburg |
| 2 | Dr. med. Volker Hagen, Medizinische Klinik II, Onkologie, St.-Johannes-Hospital, Dortmund |
| 2 | Dr. med. Enrico Schalk, Klinik für Hämatologie / Onkologie, Universitätsklinikum Magdeburg A. ö. R., Magdeburg |
| 2 | Thomas Wolff, Hämatol./Onkologische Abteilung, Hanse-Klinikum Stralsund GmbH, Stralsund |
| 2 | Prof. Dr. med. Maike de Wit, MVZ Hämatologie und Onkologie, Vivantes Netzwerk für Gesundheit, Berlin |
| 2 | Dr. med. Enno Moorahrend, Onkologische Schwerpunktpraxis, Porta Westfalica |
| 2 | Dr. med. Heinz Kirchen, I. Medizinische Abteilung, Krankenhaus der Barmherzigen Brüder, Trier |
| 2 | Dr. med. Jürgen Anhuf, Dr. Selbach, Dr. Anhuf, GbR, Duisburg |
| 2 | Dr. med. Maria Theresia Keller, Institut für Versorgungsforschung, Mayen |
| 2 | Dr. med. Manfred Reeb, Praxis, Kaiserslautern |
| 2 | Dr. med. Herbert Lebahn, Onkologisches Versorgungszentrum Friedrichshain, Berlin |
| 2 | Priv. Doz. Dr. med. Gernot Seipelt, Praxis für Internistische Onkologie, Bad Soden/Ts. |
| 2 | Dr. med. Michele Voegeli, Onkologie, Kantonsspital Liestal, Liestal, Switzerland |
| 2 | Dr. med. Stephan Bildat, Medizinische Klinik II und MVZ für Onkologie, Klinikum Kreis Herford, Herford |
| 2 | Dr. med. Michael Neise, Hämatologie und internistische Onkologie, MVZ Hämatologie und Onkologie Krefeld, Krefeld |
| 2 | Dr. med. Sven Thorspecken, Hämatologie, Onkologie, Immunologie, Klinikum Schwabing, München |
| 2 | Dr. med. Rüdiger Schnaitmann, Dr. Röddiger, Dr. Kurek, MVZ Onkologie Ostalb, Aalen |
| 2 | Dr. med. Christina Balser, Hämatologie, Gemeinschaftspraxis, Marburg |
| 2 | Prof. Dr. med. Elke Jäger, II. Medizinische Klinik, Krankenhaus Nordwest, Frankfurt |
| 2 | Dr. med. Georg Dietrich, Innere Medizin I, Krankenhaus Bietigheim, Bietigheim-Bissingen |
| 2 | Dr. med. Heike Steiniger, Praxis für Hämatologie und Onkologie Oberhausen, Oberhausen |
| 2 | Dr. med. Gerdt Hübner, Onkologische Schwerpunktpraxis, Oldenburg/Holstein |
| 2 | Dr. med. Markus Sosada, Hämatologie und Onkologie, KRH Klinikum Siloah, Hannover |
| 2 | Dr. med. Markus Ritter, Medizinische Klinik I und MVZ, Klinikverbund Südwest, Sindelfingen |
| 1 | Prof. Dr. med. Thomas Frieling, Medizinische Klinik II, Helios Klinikum Krefeld, Krefeld |
| 1 | Dr. med. Martina Stauch, Schwerpunktpraxis, Kronach |
| 1 | Priv. Doz. Dr. med. P. Markus Deckert, Med. Klinik II/ Innere, Städtisches Klinikum Brandenburg/Havel, Brandenburg a.d. Havel |
| 1 | Priv. Doz. Dr. med. Guido Trenn, Medizinische Klinik, Knappschaftskrankenhaus Bottrop, Bottrop |
| 1 | Dr. med. Gernot Reich, Medizinisches Versorgunszentrum Berlin-Tegel, Berlin |
| 1 | Dr. med. Jan Knoblich, Onco Studies Lörrach, An der Onkologie Dreiländereck, Lörrach |
| 1 | Dr. med. Gabriele Käfer, Innere Medizin, Kreiskrankenhaus Sigmaringen, Sigmaringen |
| 1 | Dr. med. Claudia Spohn, Gemeinschaftspraxis, Halle |
| 1 | Dr. med. Henry Simon, Innere Medizin, Gastroenterologie und Tumormedizin, medius Kliniken gGmbH, Ostfildern/ Ruit |
| 1 | Dr. med. Hans-Roland Schmitt, Praxis, Gerlingen |
| 1 | Dr. med. Kerstin Stahlhut, Innere Medizin, Immanuel Klinik Rüdersdorf, Rüdersdorf |
| 1 | Prof. Dr. med. Markus Ruhnke, Hämatologie/ Onkologie, Paracelsus Klinik Osnabrück, Osnabrück |
| 1 | Dr. med. Stefan Wilhelm, Onkologische Gemeinschaftspraxis, Güstrow |
| 1 | Dr. med. Michael Niedermeier, Internistisches Facharztzentrum, Memmingen |
| 1 | Dr. med. Bernhard Göttler, Onkologische Gemeinschaftspraxis, Muhr am See |
| 1 | Dr. med. Hans Wilhelm Dübbers, Dr. Burstedde, Überörtliche Gemeinschaftspraxis, Ahaus |
| 1 | Prof. Dr. med. Hans-Josef Weh, Med. Klinik II Abt. Hämatologie/Onkologie, St. Franziskus Hospital, Bielefeld |
| 1 | Dr. med. Karl Murmann, MVZ Klinikum Straubing GmbH, Straubing |
| 1 | Dr. med. Michael Klein, Medizinische Klinik I, Prosper-Hospital, Recklinghausen |
| 1 | Barbara Kempf, Medizinische Klinik III, Klinikum Landshut, Landshut |
| 1 | Prof. Dr. med. Anke Claudia Reinacher-Schick, Abteilung für Hämatologie, Onkologie - Studienambulanz, St. Josef-Hospital - Klinikum der Ruhr-Universität, Bochum |
| 1 | Dr. med. Holger Schulz, pioh Frechen, Frechen |
| 1 | Dr. med. Dirk Tummes, Gemeinschaftspraxis, Aachen |
| 1 | Prof. Dr. med. Wolff Schmiegel, Medizinische Klinik, Knappschaftskrankenhaus Bochum, Bochum |
| 1 | Dr. med. C. Priebe-Richter, Hämatologisch-Onkologische Praxis, Stadthagen |
| 1 | Christina Levknecht, Internistische Gemeinschaftspraxis, Detmold |
| 1 | Dr. med. Frank Heits, I. Med. Klinik, Diakoniekrankenhaus, Rotenburg/ Wümme |
| 1 | Dr. med. Christoph Sick, Onkologische Schwerpunktpraxis, Bremen |
| 1 | Dr. med. Ingo Zander, Gemeinschaftspraxis, Hannover |
| 1 | Prof. Dr. med. Kai Neben, MVZ Onkologie / Medizinische Klinik 2, Stadtklinik Baden-Baden, Baden-Baden |
| 1 | Dr. med. Stefan Schütz, Gemeinschaftspraxis, Bremerhaven |
| 1 | Dr. med. Norbert Grobe, Innere Med./Hämatol. Abt., Dietrich-Bonhoeffer-Klinikum, Neubrandenburg |
| 1 | Dr. med. Lothar Schulz, Innere Medizin, Klinikum Garmisch-Partenkirchen, Garmisch-Partenkirchen |
| 1 | Dr. med. Manfred Welslau, phase drei Hämato-Onkologischer Studienkreis, am Klinikum Aschaffenburg, Aschaffenburg |
| 1 | Dr. med. Christiane Hinske, Onkologische Gemeinschaftspraxis Würselen, Würselen |
| 1 | Dr. med. Dietmar Reichert, Hämatologie und Internistischen Onkologie, Ubbo-Emmius Klinik, Aurich |
| 1 | Dr. med. Peter Ehscheidt, Praxis Innere Medizin/Hämatologie/Onkologie, Neuwied |
| 1 | Dr. med. Veronika Ballova, Onkologie KSB, Kantonsspital Baden, Baden, Switzerland |
| 1 | Dr. Xaver Schiel, Tumorzentrum München Süd, Klinikum Harlaching, München |
| 1 | Dr. med. Andrea Sendler, Medizinische Klinik III, Klinikum Hanau gGmbH, Hanau |
| 1 | Priv. Doz. Dr. med. Christof Lamberti, V. Medizinische Klinik, Klinikum Coburg, Coburg |
| 1 | Carla Hannig, Gemeinschaftspraxis, Bottrop |
| 1 | Dr. med. Lutz Jakobasch, Gemeinschaftspraxis, Dresden |
| 1 | Dr. med. Arabella Konstanze Lechner, Internistische Gemeinschaftspraxis, Nürnberg |
| 1 | Dr. med. Sven Detken, Helios MVZ Südniedersachsen GmbH, Northeim |
| 1 | Dr. med. Gabriele Doering, Onkologische Praxis, Bremen |
| 1 | Dr. med. Robert Funck, MVZ Onkologie, Klinikum Weiden, Weiden |
| 1 | Dr. med. Annkristin Heine, Medizinische Klinik III, Universitätsklinik Bonn, Bonn |
| 1 | Dr. med. Torsten Kamp, Hämat.-Onkologische Gemeinschaftspraxis, Wendlingen |
| 1 | Dr. med. Eray Gökkurt, MVZ für Innere Medizin, Hamburg |
| 1 | Dr. med. Bernd Schönberger, MVZ Onkologie Rehling, Rehling |
| 1 | Dr. med. Sabine Hahnfeld, Praxis für Innere Medizin und Onkologie, Jena |
